# Supplementary material for: Engineering tumor-specific gene nanomedicine to recruit and activate T cells for enhanced immunotherapy
Source: Nat Commun. 2023 Apr 8;14:1993. doi: 10.1038/s41467-023-37656-w (PMC10082825; doi:10.1038/s41467-023-37656-w)
Supplement: Supplementary file 1 — Supplementary Information [file 41467_2023_37656_MOESM1_ESM.pdf]

Supplementary Information

Engineering tumor-specific gene nanomedicine to recruit and activate T cells for enhanced immunotherapy

Yue Wang[#,1], Shi-Kun Zhou[#,1], Yan Wang[2], Zi-Dong Lu[2], Yue Zhang[1,3], Cong-Fei Xu*[,1,3,4], Jun Wang*[,1,3,5]

[1]School of Biomedical Sciences and Engineering, South China University of Technology, Guangzhou International Campus, Guangzhou 511442, P.R. China

[2]School of Medicine, South China University of Technology, Guangzhou 510006, P.R. China

[3]National Engineering Research Center for Tissue Restoration and Reconstruction, South China University of Technology, Guangzhou 510006, P.R. China

[4]Guangdong Provincial Key Laboratory of Biomedical Engineering, South China University of Technology, Guangzhou 510006, P.R. China

[5]Key Laboratory of Biomedical Materials and Engineering of the Ministry of Education, South China University of Technology, Guangzhou 510006, P.R. China

[#]These authors contributed equally: Yue Wang, Shi-Kun Zhou.

***Corresponding author:** Cong-Fei Xu, xucf@scut.edu.cn, Jun Wang, mcjwang@scut.edu.cn

Content

Supplementary Figure 1 Construction of pTyr-C9AP, pSur-C9AP and other control plasmids.

Supplementary Figure 2 Stability of $NP_{Tyr\text{-}C9AP}$.

Supplementary Figure 3 mRNA expression of CXCL9 and αPD-L1 in B16-F10 cells and the other 7 types of cells after $NP_{Tyr\text{-}C9AP}$ transfection.

Supplementary Figure 4 Organ distribution of $NP_{Tyr\text{-}C9AP}$ in bilateral tumor model.

Supplementary Figure 5 mRNA expression of CXCL9 and αPD-L1 in $NP_{Tyr\text{-}C9AP}$-transfected B16-F10 cells.

Supplementary Figure 6 Representative flow cytometry plots of $CD8^+$ T cells recruited into the lower chamber of the transwell plates.

Supplementary Figure 7 Western blot analysis of αPD-L1 expression in $NP_{Tyr\text{-}C9AP}$-transfected B16-F10 cells.

Supplementary Figure 8 Representative flow cytometry plots of PD-L1 expression of IFN-γ-stimulated-B16-F10 cells.

Supplementary Figure 9 ELISA analysis of the capability of αPD-L1 to bind PD-L1.

Supplementary Figure 10 mRNA expression of CXCL9 and αPD-L1 in B16-F10 melanoma after different treatments.

Supplementary Figure 11 Serum concentrations of CXCL9 after different treatments.

Supplementary Figure 12 Western blot analysis of αPD-L1 expression in B16-F10 melanoma after different treatments.

Supplementary Figure 13 Body weights of mice bearing B16-F10 melanoma after different treatments.

Supplementary Figure 14 Representative H&E staining of major organs after different treatments.

Supplementary Figure 15 Potential liver toxicity analysis of $NP_{Tyr\text{-}C9AP}$.

Supplementary Figure 16 $NP_{Tyr\text{-}C9AP}$ induces melanoma-specific therapeutic effects.

Supplementary Figure 17 Gating strategies of flow cytometry analysis of immune cells in B16-F10 melanoma.

Supplementary Figure 18 Percentages of $CD3^+$ T cells in B16-F10 melanoma after different treatments.

Supplementary Figure 19 Representative flow cytometry plots of $CD8^+$ T cells and CD69-positive $CD8^+$ T cells in B16-F10 melanoma after different treatments.

Supplementary Figure 20 Percentages of granzyme B-, perforin-, IFN-γ-positive $CD8^+$ T cells in B16-F10 melanoma after different treatments.

Supplementary Figure 21 Representative flow cytometry plots of NK cells, macrophages, MDSCs and Tregs in B16-F10 melanoma after different treatments.

Supplementary Figure 22 qRT–PCR analysis of the cytokine changes in tumor microenvironment after different treatments.

Supplementary Figure 23 Body weights of mice bearing B16-F10 melanoma after different treatments.

Supplementary Figure 24 Representative flow cytometry plots of CD69-, granzyme B-, perforin- and IFN-γ-positive $CD8^+$ T cells in B16-F10 melanoma after different treatments.

Supplementary Figure 25 Ratios of $CD8^+$ T cells/Tregs, and representative flow cytometry plots of $CD8^+$ T cells and Tregs in B16-F10 melanoma after different treatments.

Supplementary Figure 26 Concentrations of CXCL9 and αPD-L1 in the culture supernatants of different melanoma cells after $NP_{Tyr\text{-}C9AP}$ transfection.

Supplementary Figure 27 Final tumor weights of Clone M-3 and YUMM1.7 melanoma-bearing mice after $NP_{Tyr\text{-}C9AP}$ treatments.

Supplementary Figure 28 Representative flow cytometry plots of $CD8^+$ T cells and CD69-positive $CD8^+$ T cells in different melanomas after $NP_{Tyr\text{-}C9AP}$ treatments.

Supplementary Figure 29 Concentrations of CXCL9 and αPD-L1 proteins in different tumors and normal organs after $NP_{Sur\text{-}C9AP}$ treatments.

Supplementary Figure 30 Representative flow cytometry plots of $CD3^+$ T cell percentages in $CD45^+$ cells, and numbers of $CD3^+$ T cells in B16-F10 melanoma after $NP_{Sur\text{-}C9AP}$ treatments.

Supplementary Figure 31 Representative flow cytometry plots of $CD8^+$ T cells, CD69-positive $CD8^+$ T cells, Tregs and granzyme B-, perforin-, IFN-γ-positive $CD8^+$ T cells in B16-F10 melanoma after $NP_{Sur\text{-}C9AP}$ treatments.

Supplementary Figure 32 Final tumor weights of CT26, Panc02 and 4T1 tumor-bearing mice after $NP_{Sur\text{-}C9AP}$ treatments.

Supplementary Figure 33 Representative flow cytometry plots of $CD3^+$ T cells and CD69-positive $CD8^+$ T cells in different tumors after $NP_{Sur\text{-}C9AP}$ treatments.

Supplementary Table 1 DNA sequences of Tyr promoter, Sur promoter, CXCL9 and αPD-L1.

Supplementary Table 2 qRT-PCR primer sequences for analyzing the expression of CXCL9 and αPD-L1.

Supplementary Table 3 qRT-PCR primer sequences for analyzing the expression of 5 key cytokines.

Supplementary Figure 34 Scans of the films used to generate western blot data for Supplementary Figure 7 and Supplementary Figure 12.

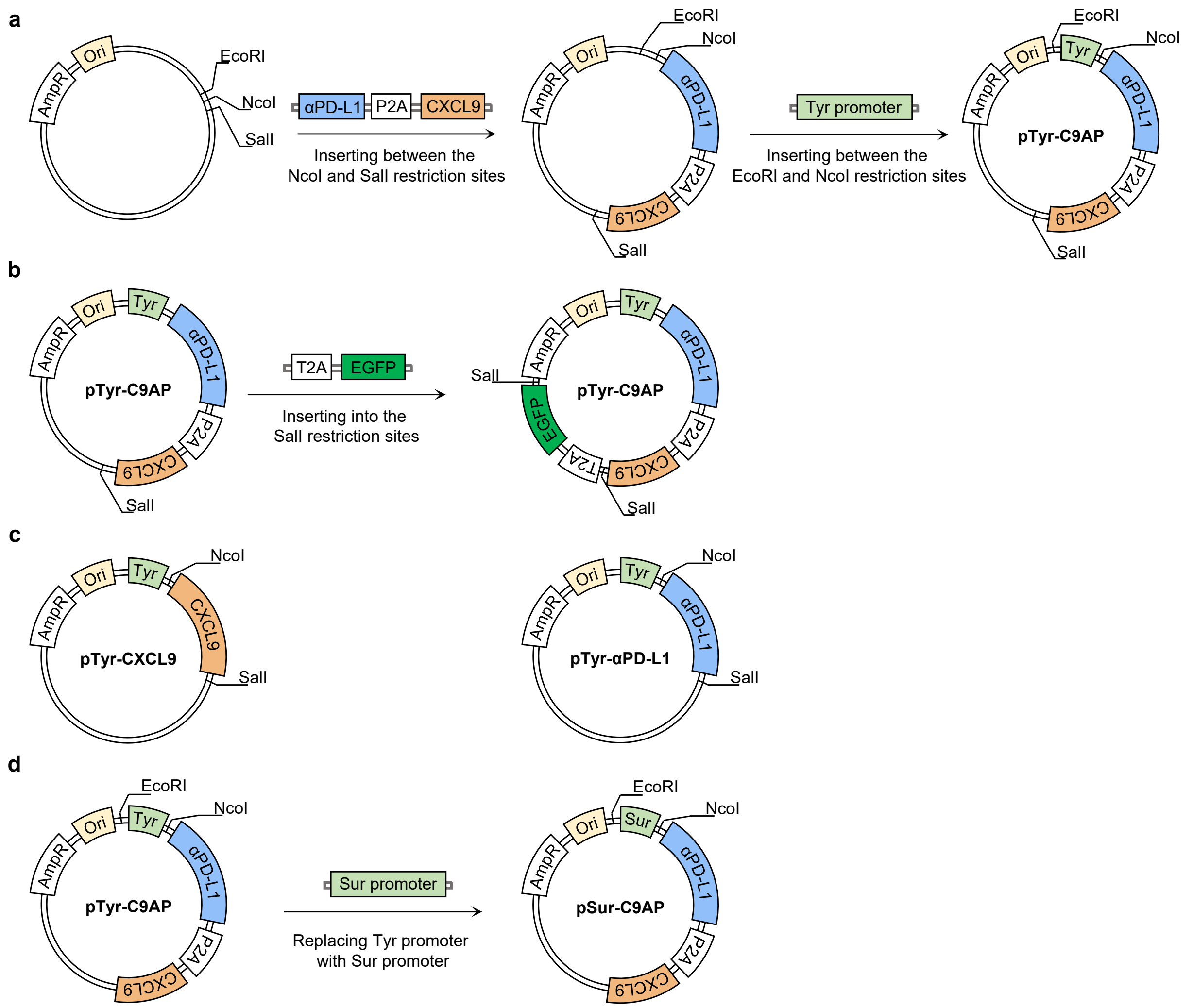

Supplementary Figure 1 Construction of pTyr-C9AP, pSur-C9AP and other control plasmids. **a** Construction of pTyr-C9AP by inserting αPD-L1-P2A-CXCL9 fragment and Tyr promoter into pUC57 backbone plasmid. pTyr-C9AP was used to co-express CXCL9 and αPD-L1 specifically in melanoma cells. **b** Construction of pTyr-C9AP containing EGFP reporter gene by inserting T2A-EGFP fragment into SalI site. pTyr-C9AP containing EGFP reporter gene was used to analyze the melanoma-specificity of $NP_{Tyr\text{-}C9AP}$. **c** Maps of pTyr-CXCL9 and pTyr-αPD-L1, which specifically express CXCL9 or αPD-L1 alone in melanoma cells, were used as the controls of pTyr-C9AP. **d** Construction of pSur-C9AP by replacing the Tyr promoter of pTyr-C9AP with Sur promoter. pSur-C9AP was used to co-express CXCL9 and αPD-L1 specifically in different tumor cells.

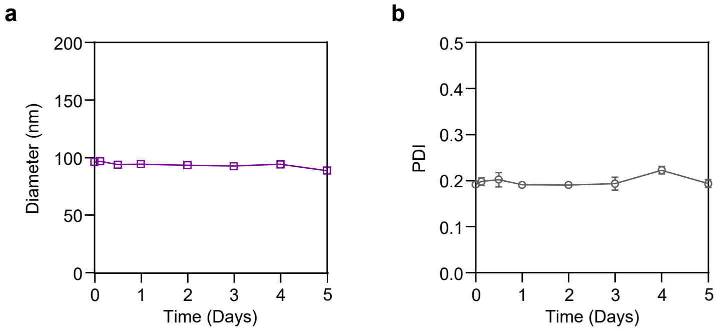

Supplementary Figure 2 Stability of NP$_{\text{Tyr-C9AP}}$. a, b Diameter (**a**) and polydispersity index (PDI) (**b**) of NP$_{\text{Tyr-C9AP}}$ after being incubated in DMEM medium containing 10% FBS. The diameter and PDI were detected by Zetasizer Nano ZS90 at different time periods. The data are shown as the means ± SEM of n = 3 independent samples. Source data are provided as a Source Data file.

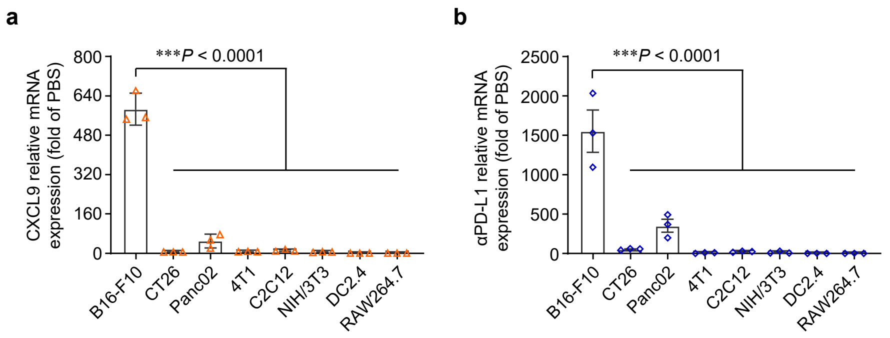

Supplementary Figure 3 mRNA expression of CXCL9 (a) and αPD-L1 (b) in B16-F10 cells and the other 7 types of cells after $NP_{Tyr\text{-}C9AP}$ transfection. B16-F10 cells and the other 7 types of cells were incubated with $NP_{Control}$ or $NP_{Tyr\text{-}C9AP}$ for 6 h, and the mRNA expression of CXCL9 and αPD-L1 were detected by qRT–PCR at 48 h after different transfections. The data are shown as the means ± SEM of $n = 3$ biologically independent samples. Statistical data were analyzed by one-way ANOVA with Tukey's multiple comparison test. ***$P < 0.001$. Source data are provided as a Source Data file.

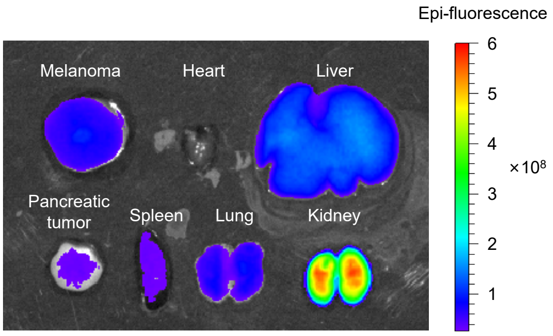

Supplementary Figure 4 Organ distribution of NP$_{Tyr\text{-}C9AP}$ in bilateral tumor model. The bilateral tumor model was established by subcutaneously inoculating 1.5 × 10^6 YUMM1.7 melanoma cells into the right flank of C57BL/6 mice and 5 × 10^6 Panc02 pancreatic tumor cells into the left flank. When the melanoma volume reached 400–500 mm^3, the mice were intravenously injected Cy5-labeled NP$_{Tyr\text{-}C9AP}$ at a dose of 1 mg Cy5-labeled pTyr-C9AP per kg body weight. Twelve hours later, the YUMM1.7 melanoma, Panc02 pancreatic tumor and normal organs were collected, and were analyzed using IVIS Lumina III Living Image system (PerkinElmer, MA, USA). The data are representative of three biologically independent mice.

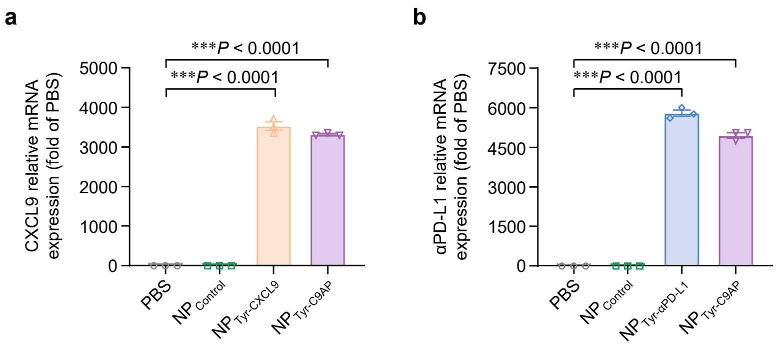

Supplementary Figure 5 mRNA expression of CXCL9 and αPD-L1 in $NP_{Tyr\text{-}C9AP}$-transfected B16-F10 cells. a B16-F10 cells were incubated with PBS, $NP_{Control}$, $NP_{Tyr\text{-}CXCL9}$ or $NP_{Tyr\text{-}C9AP}$ for 6 h, and CXCL9 mRNA expression was detected by qRT–PCR at 48 h after different transfections. **b** B16-F10 cells were incubated with PBS, $NP_{Control}$, $NP_{Tyr\text{-}\alpha PD\text{-}L1}$ or $NP_{Tyr\text{-}C9AP}$ for 6 h, and αPD-L1 mRNA expression was detected by qRT–PCR at 48 h after different transfections. The data are shown as the means ± SEM of $n = 3$ biologically independent samples. Statistical data were analyzed by one-way ANOVA with Tukey's multiple comparison test. $***P < 0.001$. Source data are provided as a Source Data file.

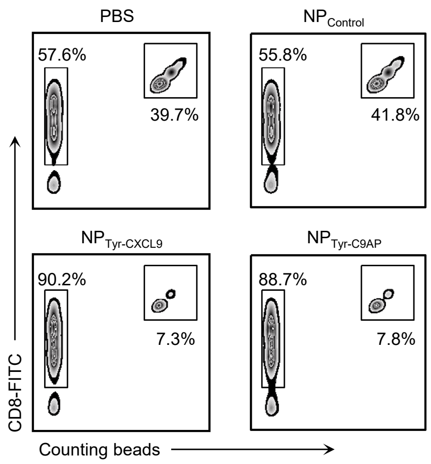

Supplementary Figure 6 Representative flow cytometry plots of CD8$^+$ T cells recruited into the lower chamber of the transwell plates. Activated CD8$^+$ T cells were labeled with DiI dye, and were cultured in the upper chamber of transwell plates at the density of 1 × 10^6 cells per well. Then, the culture supernatants of PBS, NP$_{Control}$, NP$_{Tyr-CXCL9}$ or NP$_{Tyr-C9AP}$-transfected B16-F10 cells were placed in the lower chamber. Three hours after incubation, CD8$^+$ T cells recruited in the lower chamber were collected and were labeled with FITC anti-mouse CD8a antibody. Then, 10 μl of Precision Count Beads were added into each sample before being analyzed by flow cytometry. The numbers of recruited CD8$^+$ T cells were calculated using the formula: $X = Y \times Z$ (X, absolute count of CD8$^+$ T cells; Y, ratio of CD8$^+$ T cells/counting beads; Z, absolute count of counting beads). The data are representative of three biologically independent samples.

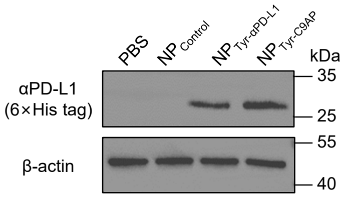

Supplementary Figure 7 Western blot analysis of αPD-L1 expression in $NP_{Tyr\text{-}C9AP}$-transfected B16-F10 cells. B16-F10 cells were incubated with PBS, $NP_{Control}$, $NP_{Tyr\text{-}\alpha PD\text{-}L1}$ or $NP_{Tyr\text{-}C9AP}$ for 6 h, and the culture supernatants were collected to detect αPD-L1 expression by western blot at 72 h after different transfections. The expression of β-actin in the cell lysates was detected as the reference control. The data are representative of three independent experiments. Source data are provided as a Source Data file.

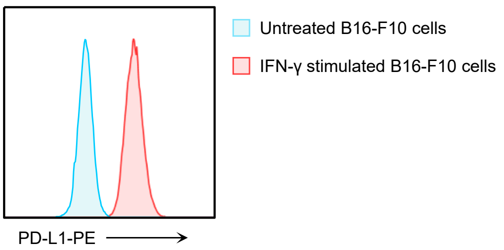

Supplementary Figure 8 Representative flow cytometry plots of PD-L1 expression of IFN-γ-stimulated-B16-F10 cells. B16-F10 cells were stimulated with IFN-γ (20 ng ml^{-1}) for 24 h. Then, the IFN-γ-stimulated B16-F10 cells were stained with PE anti-mouse PD-L1 antibody (clone: 10F.9G2, BioLegend), and the expression of PD-L1 was analyzed by flow cytometry. The data are representative of three biologically independent samples.

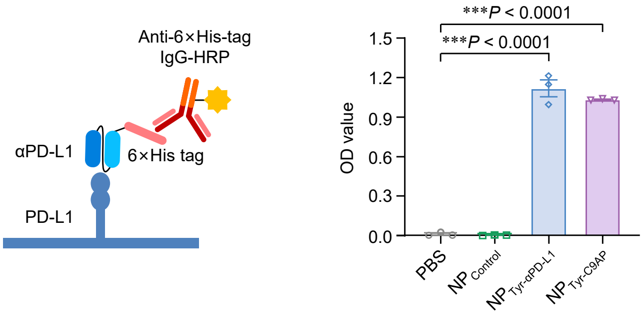

Supplementary Figure 9 ELISA analysis of the capability of αPD-L1 to bind PD-L1. The culture supernatants of B16-F10 cells transfected with PBS, $NP_{Control}$, $NP_{Tyr\text{-}\alpha PD\text{-}L1}$ or $NP_{Tyr\text{-}C9AP}$ were collected at 72 h after different transfections. Then, the culture supernatants were added into the ELISA plates coated with PD-L1 molecule (SinoBiological, Beijing, China). One hour after incubation, the quantity of αPD-L1 that bound to PD-L1 molecule was detected using anti-6×His tag IgG-HRP (SinoBiological) to label the 6×His tag of αPD-L1. The data are shown as the means ± SEM of $n = 3$ biologically independent samples. Statistical data were analyzed by one-way ANOVA with Tukey's multiple comparison test. ***$P < 0.001$. Source data are provided as a Source Data file.

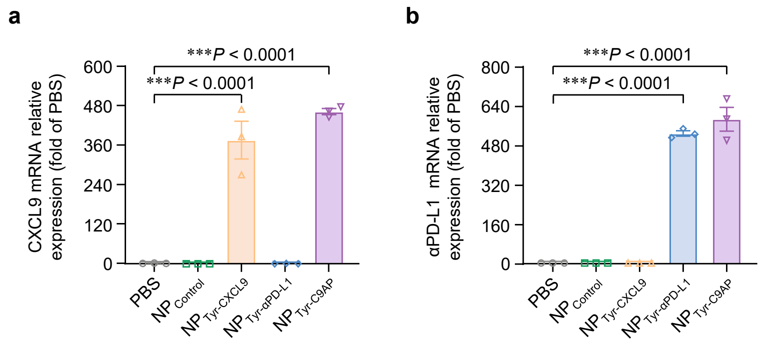

Supplementary Figure 10 mRNA expression of CXCL9 (a) and αPD-L1 (b) in B16-F10 melanoma after different treatments. Mice bearing B16-F10 melanoma were intravenously injected with PBS, $NP_{Control}$, $NP_{Tyr\text{-}CXCL9}$, $NP_{Tyr\text{-}\alpha PD\text{-}L1}$ or $NP_{Tyr\text{-}C9AP}$ every other day for three injections (n = 3 mice per group). The injection doses were 1 mg pUC57, pTyr-CXCL9, pTyr-αPD-L1 or pTyr-C9AP per kg body weight. Seventy-two hours after injection, the mRNA expression of CXCL9 and αPD-L1 in melanoma tissues were detected by qRT–PCR. The data are shown as the means ± SEM of n = 3 biologically independent mice. Statistical data were analyzed by one-way ANOVA with Tukey's multiple comparison test. ***P < 0.001. Source data are provided as a Source Data file.

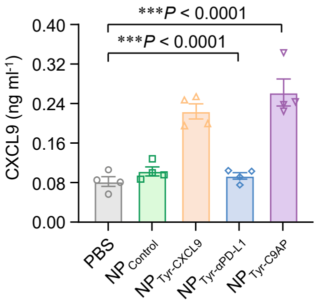

Supplementary Figure 11 Serum concentrations of CXCL9 after different treatments. Mice bearing B16-F10 melanoma were intravenously injected with PBS, $NP_{Control}$, $NP_{Tyr\text{-}CXCL9}$, $NP_{Tyr\text{-}\alpha PD\text{-}L1}$ or $NP_{Tyr\text{-}C9AP}$ every other day for three injections (n = 4 mice per group). The injection doses were 1 mg pUC57, pTyr-CXCL9, pTyr-αPD-L1 or pTyr-C9AP per kg body weight. Seventy-two hours after injection, the serum samples were collected, and the concentrations of CXCL9 in the serum were detected by ELISA. The data are shown as the means ± SEM of n = 4 biologically independent mice. Statistical data were analyzed by one-way ANOVA with Tukey's multiple comparison test. ***$P < 0.001$. Source data are provided as a Source Data file.

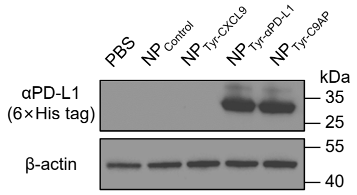

Supplementary Figure 12 Western blot analysis of αPD-L1 expression in B16-F10 melanoma after different treatments. Mice bearing B16-F10 melanoma were intravenously injected with PBS, $NP_{Control}$, $NP_{Tyr\text{-}CXCL9}$, $NP_{Tyr\text{-}\alpha PD\text{-}L1}$ or $NP_{Tyr\text{-}C9AP}$ every other day for three injections (*n* = 4 mice per group). The injection doses were 1 mg pUC57, pTyr-CXCL9, pTyr-αPD-L1 or pTyr-C9AP per kg body weight. Seventy-two hours after injection, the αPD-L1 expression in B16-F10 melanoma tissues were detected by western blot. The data are representative of four biologically independent mice. Source data are provided as a Source Data file.

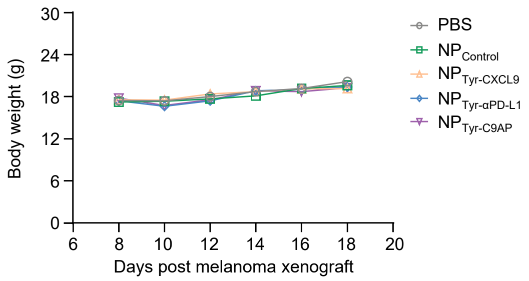

Supplementary Figure 13 Body weights of mice bearing B16-F10 melanoma after different treatments. Mice bearing B16-F10 melanoma were intravenously injected with PBS, $NP_{Control}$, $NP_{Tyr\text{-}CXCL9}$, $NP_{Tyr\text{-}\alpha PD\text{-}L1}$ or $NP_{Tyr\text{-}C9AP}$ every other day for five injections (n = 10 mice per group), as illustrated in Fig. 4c. The injection doses were 1 mg pUC57, pTyr-CXCL9, pTyr-αPD-L1 or pTyr-C9AP per kg body weight. The body weights of the mice were monitored every other day since the first injection. The data are shown as the means ± SEM of n = 10 biologically independent mice. Source data are provided as a Source Data file.

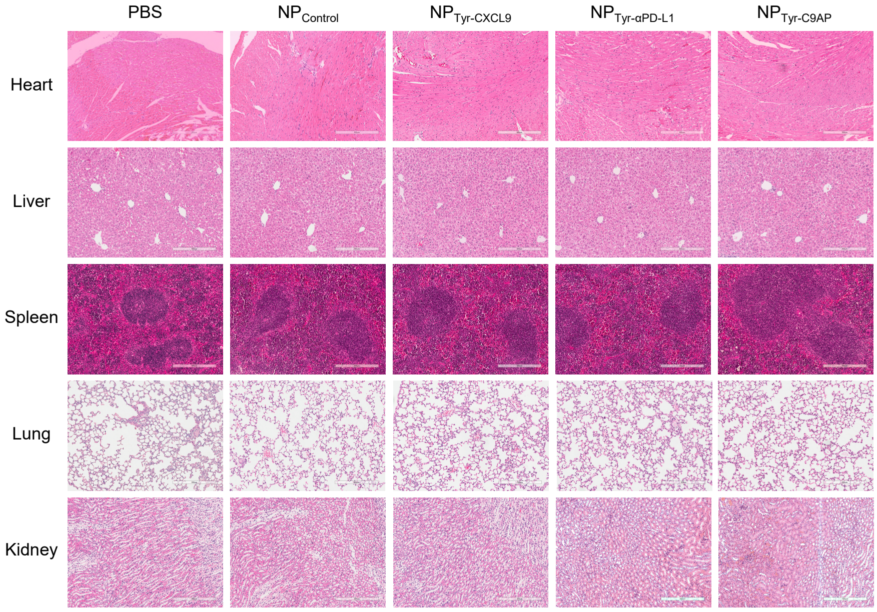

Supplementary Figure 14 Representative H&E staining of major organs after different treatments. The major organs (including heart, liver, spleen, lung and kidney) of mice bearing B16-F10 melanoma were collected at the end of different treatments, and the histopathology of each organ was analyzed by haemotoxylin & eosin (H&E) staining. The data were collected using Digital Pathology Scanner software of Digital pathology scanning system Aperio CS2 (Leica, NJ, USA). Scale bar = 300 μm. The data are representative of four biologically independent mice.

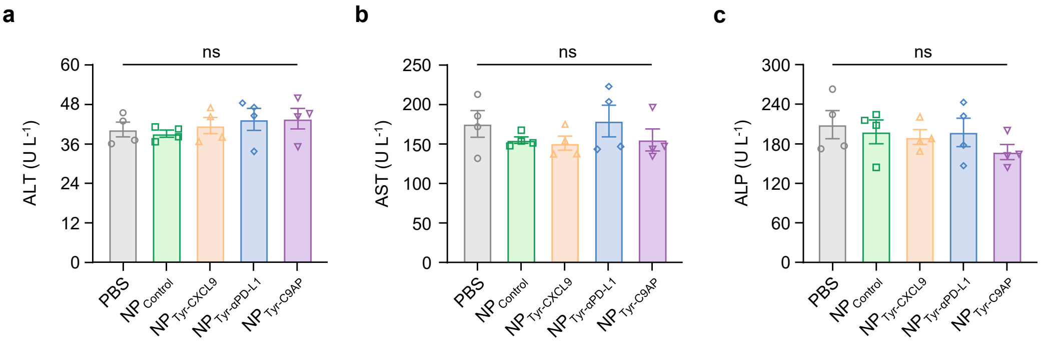

Supplementary Figure 15 Potential liver toxicity analysis of NP$_{\text{Tyr-C9AP}}$. a to **c** Serum levels of alanine aminotransferase (ALT) (**a**), aspartate aminotransferase (AST) (**b**) and alkaline phosphatase (ALP) (**c**) after different treatments. C57BL/6 mice were intravenously injected with PBS, NP$_{\text{Control}}$, NP$_{\text{Tyr-CXCL9}}$, NP$_{\text{Tyr-αPD-L1}}$ or NP$_{\text{Tyr-C9AP}}$ every other day for five injections (n = 4 mice per group). The injection doses were 1 mg pUC57, pTyr-CXCL9, pTyr-αPD-L1 or pTyr-C9AP per kg body weight. One week after the last injection, the serum samples were collected to detect the concentrations of ALT, AST and ALP using ELISA kits (Rayto, Guangdong, China). The data are shown as the means ± SEM of n = 4 biologically independent mice. Statistical data were analyzed by one-way ANOVA with Tukey's multiple comparison test. ns indicates no significant difference. Source data are provided as a Source Data file.

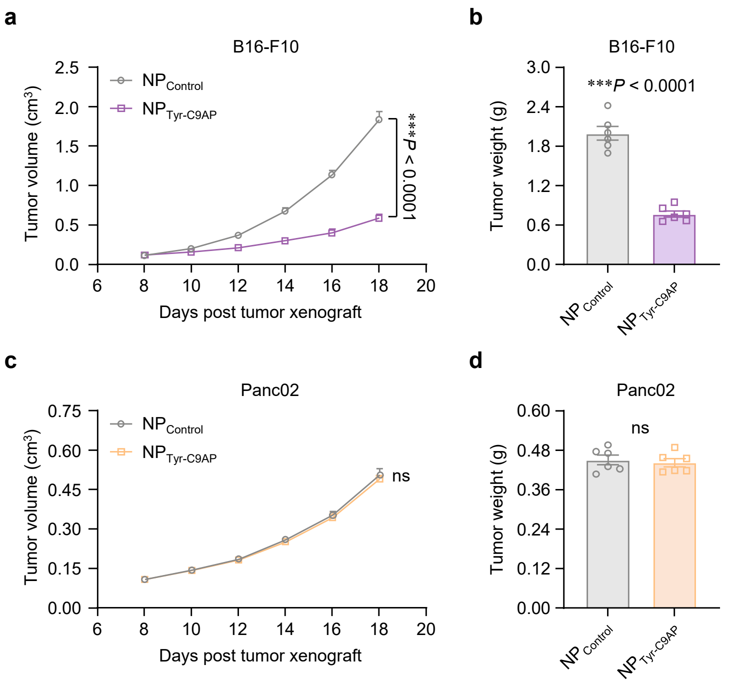

Supplementary Figure 16 $NP_{Tyr\text{-}C9AP}$ induces melanoma-specific therapeutic effects. a to **d** Growth curves and final weights of B16-F10 melanoma (**a**, **b**) and Panc02 pancreatic tumor (**c**, **d**) in the bilateral tumor-bearing mice treated with $NP_{Tyr\text{-}C9AP}$ or $NP_{Control}$ (n = 6 mice per group). Mice bearing bilateral tumors were intravenously injected with $NP_{Tyr\text{-}C9AP}$ or $NP_{Control}$ every other day for five injections. The injection doses were 1 mg pTyr-C9AP or pUC57 per kg body weight. The length and width of tumors of the mice were monitored every other day. The tumors were collected at the end of the treatments and the final tumor weights were measured. The data are shown as the means ± SEM of n = 6 biologically independent mice. Statistical data were analyzed by two-way ANOVA with the Greenhouse-Geisser correction (**a**, **c**), two-sided student's t-test (**b**, **d**). ***P < 0.001; ns indicates no significant difference. Source data are provided as a Source Data file.

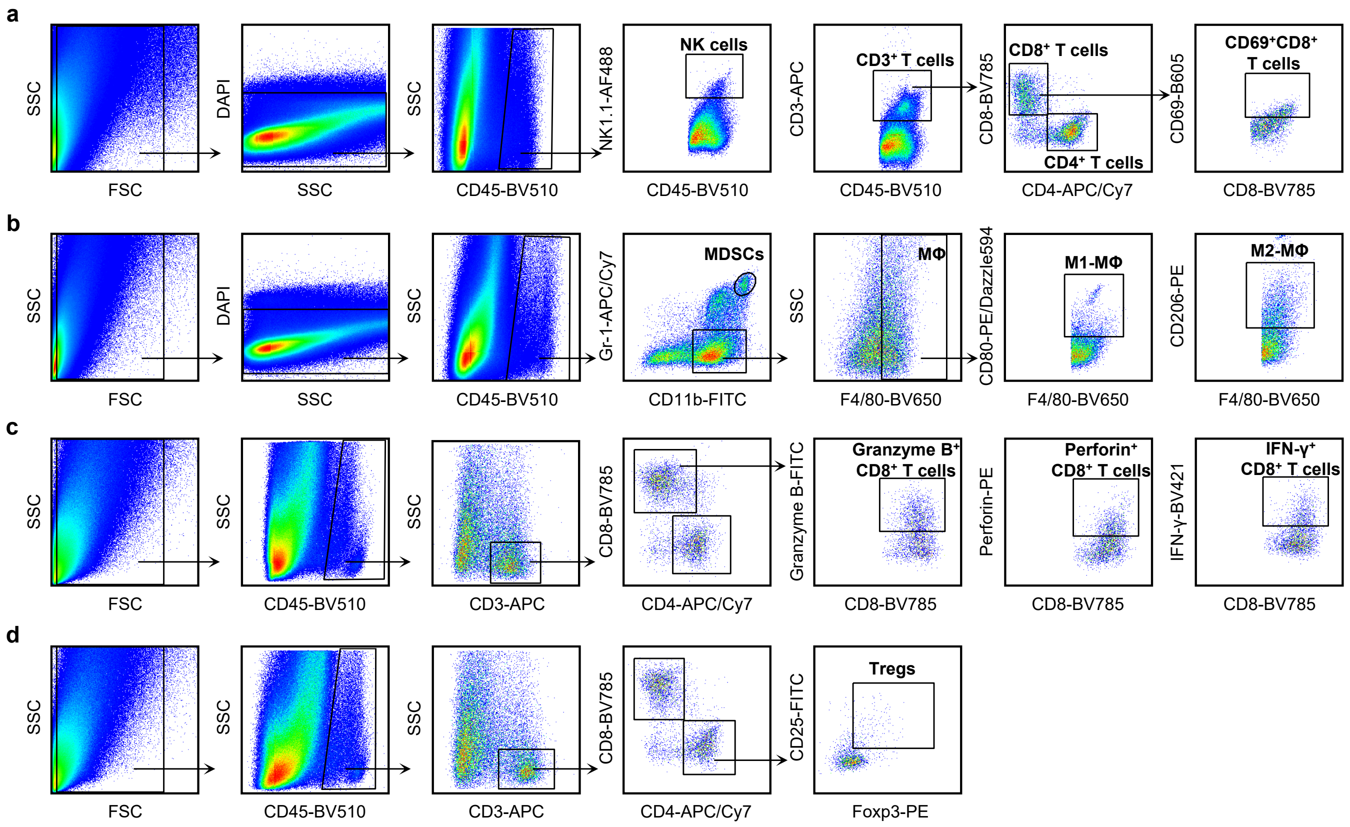

Supplementary Figure 17 Gating strategies of flow cytometry analysis of immune cells in B16-F10 melanoma. **a** Gating strategies of $CD3^+$, $CD4^+$, $CD8^+$, CD69-positive $CD8^+$ T cells and natural killer (NK) cells. **b** Gating strategies of myeloid-derived suppressor cells (MDSCs), M1-like and M2-like macrophages (MΦ). **c** Gating strategies of granzyme B-, perforin- and IFN-γ-positive $CD8^+$ T cells. **d** Gating strategies of regulatory T cells (Tregs).

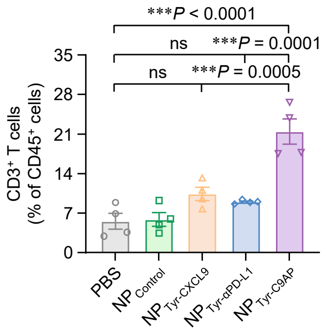

Supplementary Figure 18 Percentages of CD3$^+$ T cells in B16-F10 melanoma after different treatments. The representative flow cytometry plots were correspondingly shown in Fig. 5a. B16-F10 melanoma-bearing mice were intravenously injected with PBS, $NP_{Control}$, $NP_{Tyr\text{-}CXCL9}$, $NP_{Tyr\text{-}\alpha PD\text{-}L1}$ or $NP_{Tyr\text{-}C9AP}$ every other day for five injections. The injection doses were 1 mg pUC57, pTyr-CXCL9, pTyr-αPD-L1 or pTyr-C9AP per kg body weight. At the end of tumor treatment, the percentages of CD3$^+$ T cells in CD45$^+$ cells of melanoma were analyzed by flow cytometry. The data are shown as the means ± SEM of n = 4 biologically independent mice. Statistical data were analyzed by one-way ANOVA with Tukey's multiple comparison test. ***P < 0.001; ns indicates no significant difference. Source data are provided as a Source Data file.

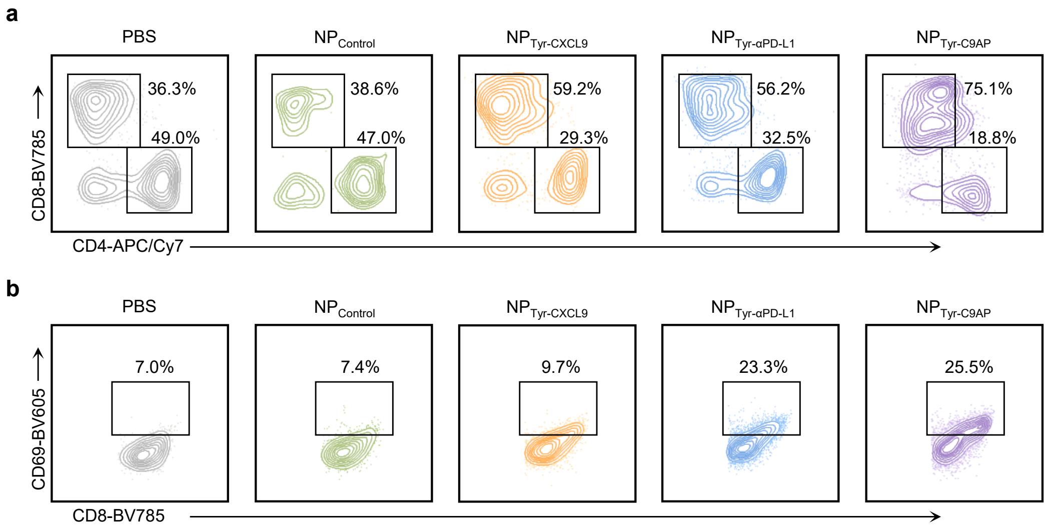

Supplementary Figure 19 Representative flow cytometry plots of CD8$^+$ T cells and CD69-positive CD8$^+$ T cells in B16-F10 melanoma after different treatments. a, b Representative flow cytometry plots of CD8$^+$ T cell percentages in CD3$^+$ T cells (**a**) and CD69-positive cell percentages in CD8$^+$ T cells (**b**) of B16-F10 melanoma. The statistical data were correspondingly shown in Fig. 5c to f.

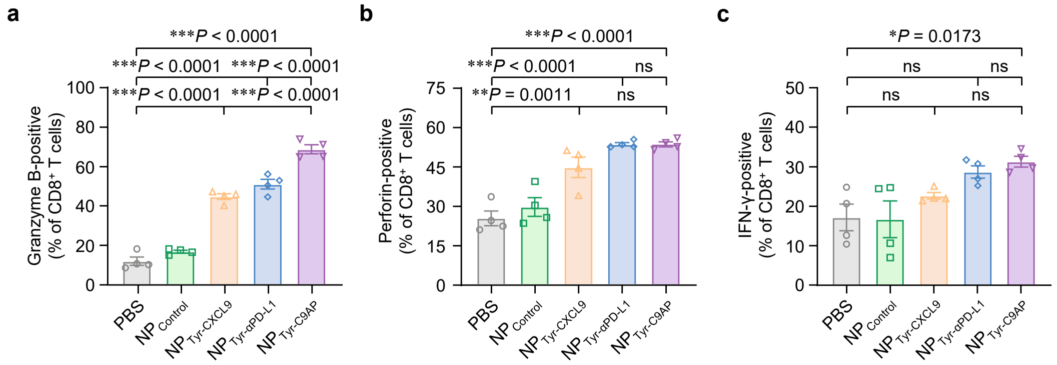

Supplementary Figure 20 Percentages of granzyme B- (a), perforin- (b), IFN-γ- (c) positive CD8$^+$ T cells in B16-F10 melanoma after different treatments. The representative flow cytometry plots were correspondingly shown in Fig. 5h to j. The data are shown as the means ± SEM of n = 4 biologically independent mice. Statistical data were analyzed by one-way ANOVA with Tukey's multiple comparison test. *P < 0.05; **P < 0.01; ***P < 0.001; ns indicates no significant difference. Source data are provided as a Source Data file.

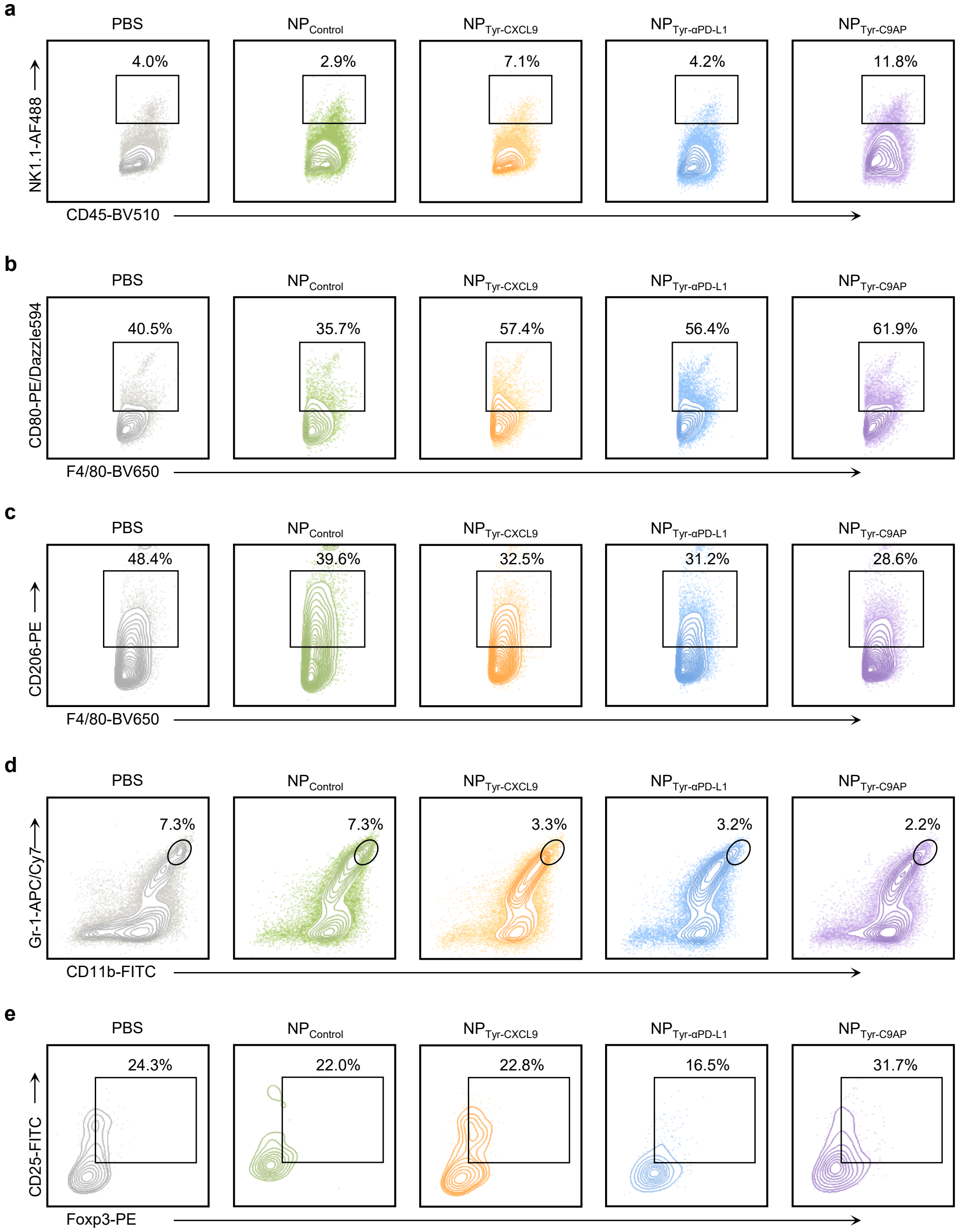

Supplementary Figure 21 Representative flow cytometry plots of NK cells, macrophages, MDSCs and Tregs in B16-F10 melanoma after different treatments. a to **e** Representative flow cytometry plots of NK cell percentages in CD45^{+} cells (**a**), M1-like macrophage percentages in macrophages (**b**), M2-like macrophage percentages in macrophages (**c**), MDSC percentages in CD45^{+} cells (**d**) and Treg percentages in CD4^{+} T cells (**e**) of B16-F10 melanoma. The statistical data were correspondingly shown in Fig. 5f, k to n.

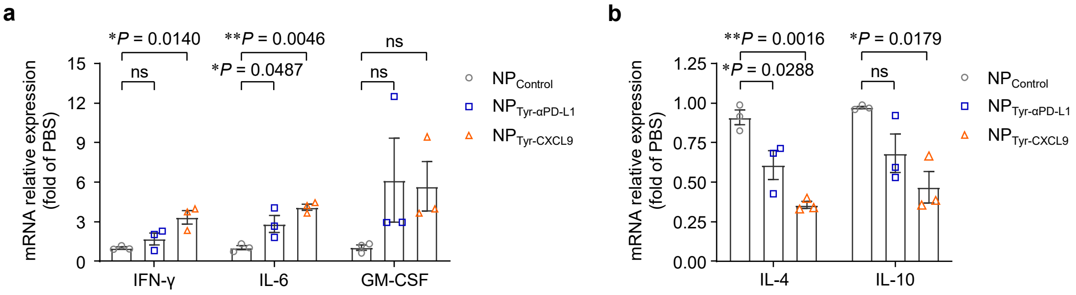

Supplementary Figure 22 qRT–PCR analysis of the cytokine changes in tumor microenvironment after different treatments. **a** mRNA expression of IFN-γ, IL-6 and GM-CSF in melanoma after different treatments. **b** mRNA expression of IL-4 and IL-10 in melanoma after different treatments. B16-F10 melanoma-bearing mice were injected with $NP_{Control}$, $NP_{Tyr\text{-}\alpha PD\text{-}L1}$ or $NP_{Tyr\text{-}CXCL9}$ every other day for five injections (n = 3 mice per group). The injection doses were 1 mg pUC57, pTyr-αPD-L1 or pTyr-CXCL9 per kg body weight. The mRNA expression of 5 key cytokines related to the transformation of immune microenvironment in melanoma were detected by qRT–PCR at the end of treatments using primers listed in Supplementary Table 3. The data are shown as the means ± SEM of n = 3 biologically independent mice. Statistical data were analyzed by one-way ANOVA with Tukey's multiple comparison test. *P < 0.05; **P < 0.01; ns indicates no significant difference. Source data are provided as a Source Data file.

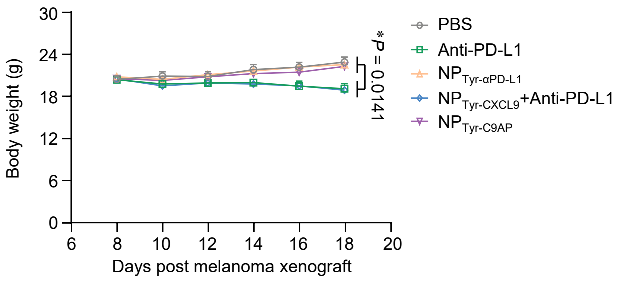

Supplementary Figure 23 Body weights of mice bearing B16-F10 melanoma after different treatments. Mice bearing B16-F10 melanoma were intravenously injected with PBS, anti-PD-L1 antibody, $NP_{Tyr-\alpha PD-L1}$, $NP_{Tyr-CXCL9}$ & anti-PD-L1 antibody or $NP_{Tyr-C9AP}$ every other day for five injections (n = 6 mice per group), as illustrated in Fig. 6a. Anti-PD-L1 antibody was injected every other day for five injections and the injection dose was 2.5 mg per kg body weight. $NP_{Tyr-CXCL9}$, $NP_{Tyr-\alpha PD-L1}$ and $NP_{Tyr-C9AP}$ were injected every other day for five injections and the injection doses were 1 mg pTyr-CXCL9, pTyr-αPD-L1 or pTyr-C9AP per kg body weight. The body weights of the mice were monitored every other day since the first injection. The data are shown as the means ± SEM of n = 6 biologically independent mice. Statistical data were analyzed by two-way ANOVA with the Greenhouse–Geisser correction. *P < 0.05. Source data are provided as a Source Data file.

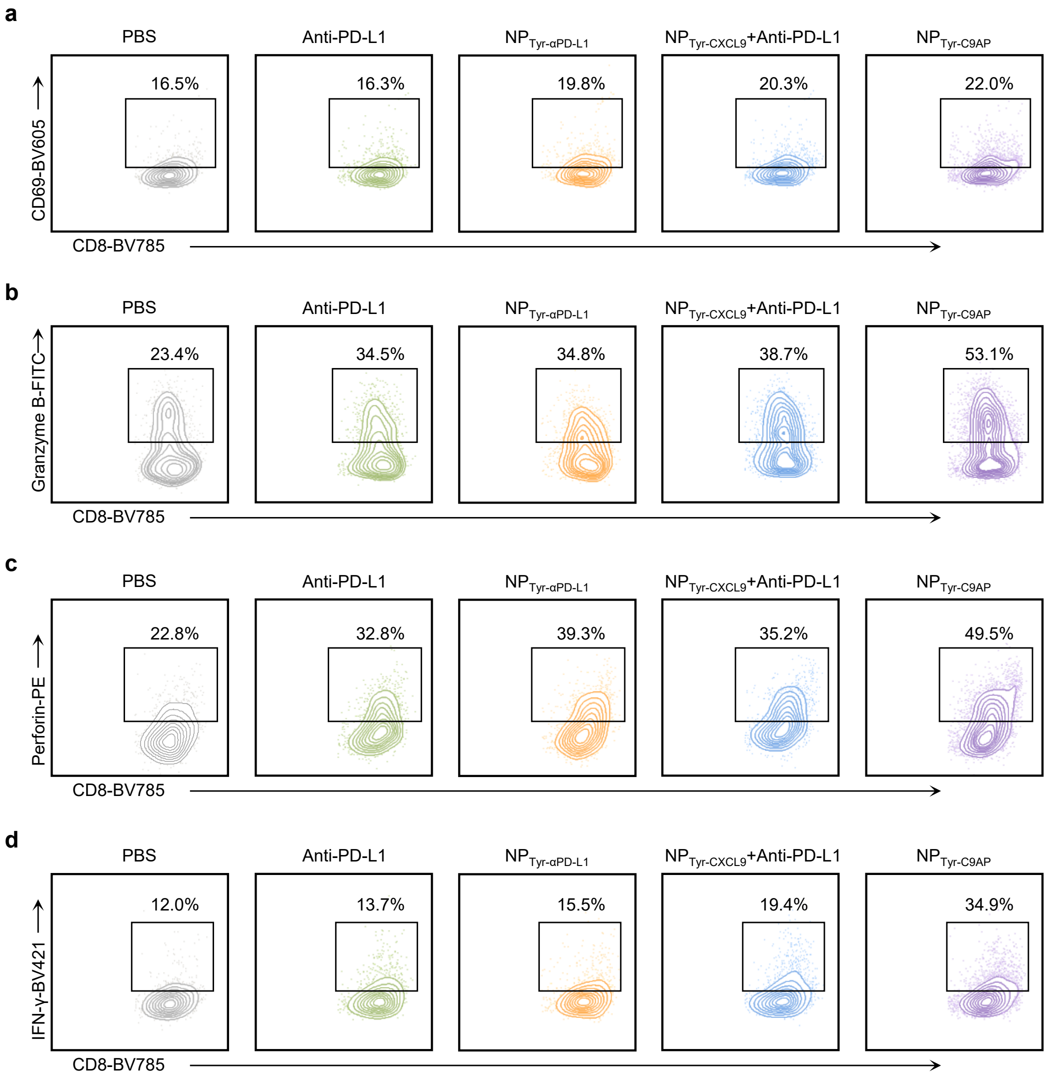

Supplementary Figure 24 Representative flow cytometry plots of CD69- (a), granzyme B- (b), perforin- (c) and IFN-γ- (d) positive CD8$^+$ T cells in B16-F10 melanoma after different treatments. The statistical data were correspondingly shown in Fig. 6f to i.

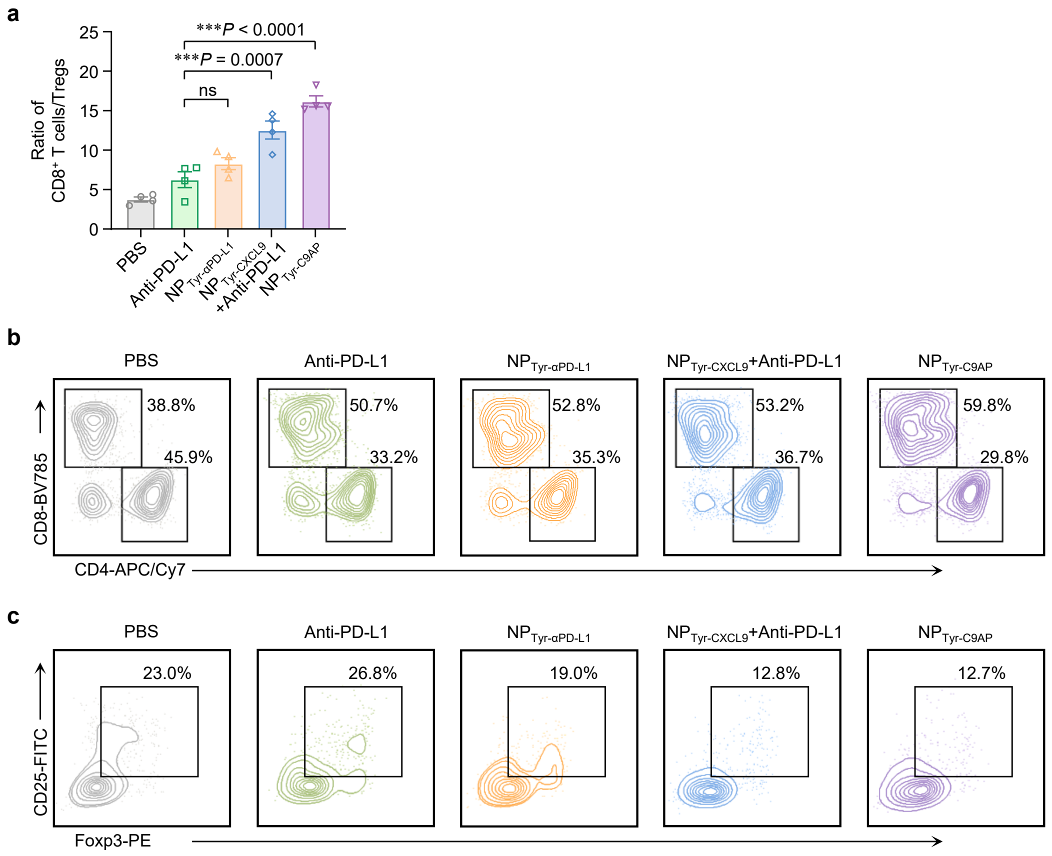

Supplementary Figure 25 Ratios of CD8$^+$ T cells/Tregs, and representative flow cytometry plots of CD8$^+$ T cells and Tregs in B16-F10 melanoma after different treatments. **a** Ratios of CD8$^+$ T cells/Tregs in B16-F10 melanoma. **b**, **c** Representative flow cytometry plots of CD8$^+$ T cell percentages in CD3$^+$ T cells (**b**) and Treg percentages in CD4$^+$ T cells (**c**) in B16-F10 melanoma. The data are shown as the means ± SEM of $n = 4$ biologically independent mice. Statistical data were analyzed by one-way ANOVA with Tukey's multiple comparison test. ***$P < 0.001$; ns indicates no significant difference. Source data are provided as a Source Data file.

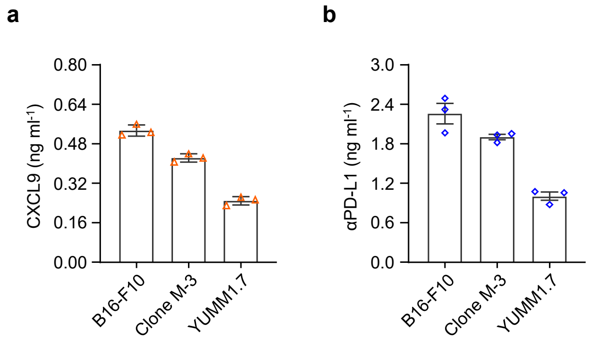

Supplementary Figure 26 Concentrations of CXCL9 (a) and αPD-L1 (b) in the culture supernatants of different melanoma cells after $NP_{Tyr\text{-}C9AP}$ transfection. B16-F10, Clone M-3 and YUMM1.7 cells were incubated with $NP_{Control}$ or $NP_{Tyr\text{-}C9AP}$ for 6 h. The transfection doses were 1 μg ml^{-1} pUC57 or pTyr-C9AP. The concentrations of CXCL9 and αPD-L1 in the culture supernatants were detected by ELISA at 72 h after different transfections. The data are shown as the means ± SEM of n = 3 biologically independent samples. Source data are provided as a Source Data file.

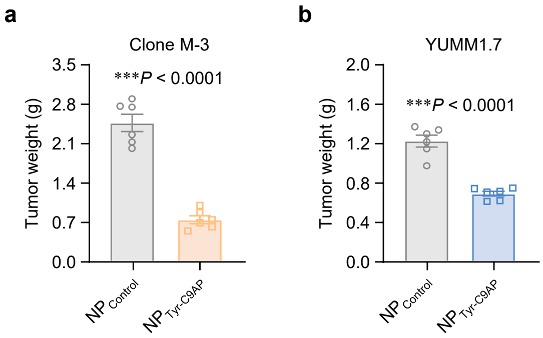

Supplementary Figure 27 Final tumor weights of Clone M-3 (a) and YUMM1.7 (b) melanoma-bearing mice after NP$_{\text{Tyr-C9AP}}$ treatments. Mice bearing Clone M-3 or YUMM1.7 melanoma were intravenously injected with NP$_{\text{Control}}$ or NP$_{\text{Tyr-C9AP}}$ every other day for five injections (n = 6 mice per group). The injection doses were 1 mg pUC57 or pTyr-C9AP per kg body weight. The final tumor weights of the different melanoma-bearing mice were measured at the end of treatments. The data are shown as the means ± SEM of n = 6 biologically independent mice. Statistical data were analyzed by two-sided student's t-test. ***$P < 0.001$. Source data are provided as a Source Data file.

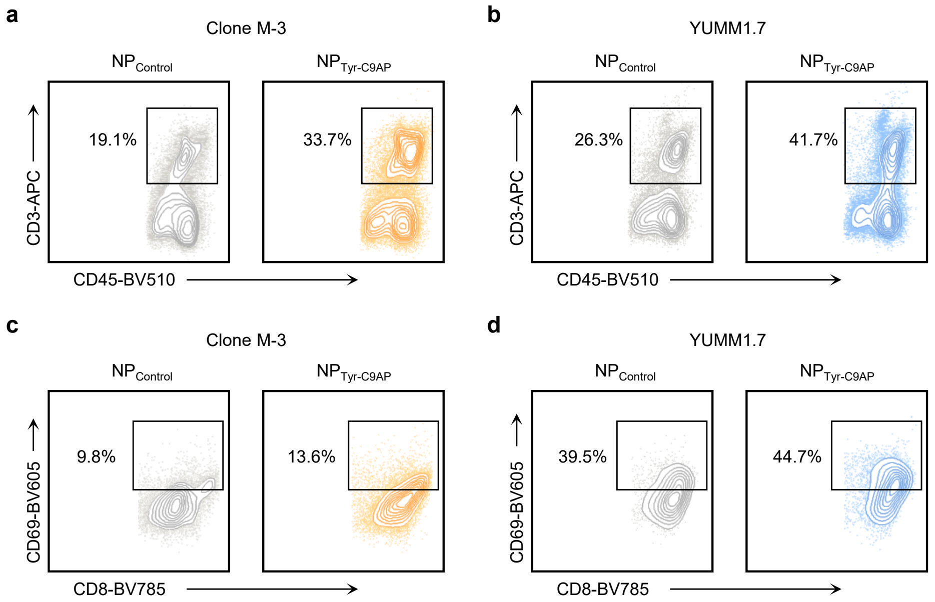

Supplementary Figure 28 Representative flow cytometry plots of CD8$^+$ T cells and CD69-positive CD8$^+$ T cells in different melanomas after NP$_{Tyr\text{-}C9AP}$ treatments. a to **d** Representative flow cytometry plots of CD3$^+$ T cell percentages in CD45$^+$ cells (**a**, **b**) and CD69-positive cell percentages in CD8$^+$ T cells (**c**, **d**) of Clone M-3 or YUMM1.7 melanoma. The statistical data were correspondingly shown in Fig. 7d, e, i, j.

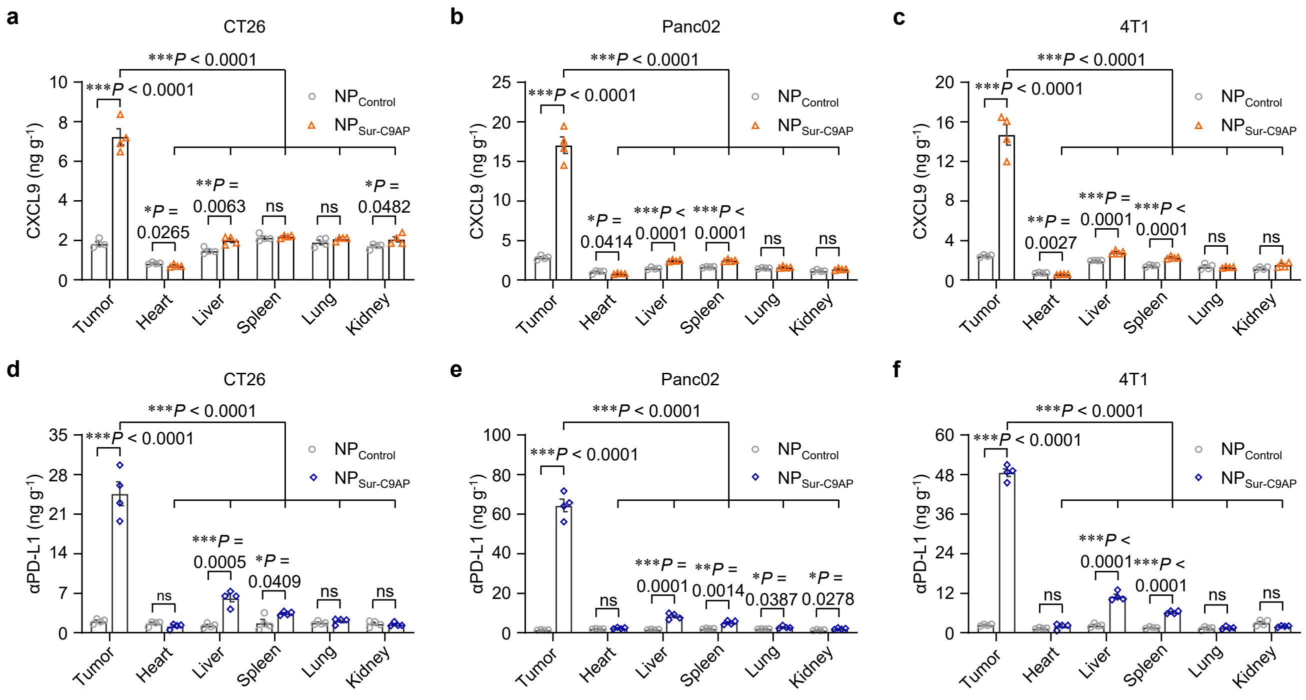

Supplementary Figure 29 Concentrations of CXCL9 and αPD-L1 proteins in different tumors and normal organs after NP$_{Sur\text{-}C9AP}$ treatments. a to **c** Concentrations of CXCL9 protein in different tumors and normal organs of mice bearing CT26 (**a**), Panc02 (**b**) or 4T1 (**c**) tumors after NP$_{Sur\text{-}C9AP}$ treatments. **d** to **f** Concentrations of αPD-L1 protein in different tumors and normal organs of mice bearing CT26 (**d**), Panc02 (**e**) or 4T1 (**f**) tumors after NP$_{Sur\text{-}C9AP}$ treatments. Mice bearing CT26, Panc02 or 4T1 tumors were intravenously injected with NP$_{Control}$ or NP$_{Sur\text{-}C9AP}$ every other day for three injections (n = 4 mice per group). The injection doses were 1 mg pUC57 or pSur-C9AP per kg body weight. Seventy-two hours after the last injection, the tumor tissue, liver, lung, spleen, kidney and heart were isolated to detect the concentrations of CXCL9 and αPD-L1 using ELISA. The concentrations of CXCL9 and αPD-L1 were indicated as ng protein per gram tissue (ng g^{-1}). The data are shown as the means ± SEM of n = 4 biologically independent mice. Statistical data were analyzed by one-way ANOVA with Tukey's multiple comparison test, two-sided student's t-test. *P < 0.05; **P < 0.01; ***P < 0.001; ns indicates no significant difference. Source data are provided as a Source Data file.

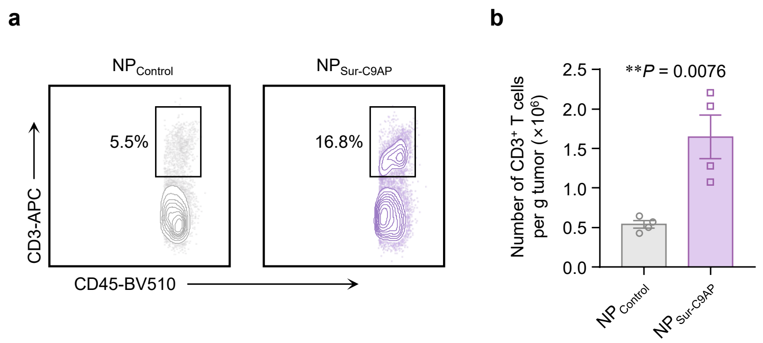

Supplementary Figure 30 Representative flow cytometry plots of $CD3^+$ T cell percentages in $CD45^+$ cells (a), and numbers of $CD3^+$ T cells (b) in B16-F10 melanoma after $NP_{Sur-C9AP}$ treatments. Mice bearing B16-F10 melanoma were intravenously injected with $NP_{Control}$ or $NP_{Sur-C9AP}$ every other day for five injections (n = 4 mice per group). The injection doses were 1 mg pUC57 or pSur-C9AP per kg body weight. At the end of tumor treatment, the percentages of $CD3^+$ T cells in $CD45^+$ cells of melanoma were analyzed by flow cytometry. The statistical data of $CD3^+$ T cell percentages were correspondingly shown in Fig. 8g. The data are shown as the means ± SEM of n = 4 biologically independent mice. Statistical data were analyzed by two-sided student's t-test. $^{**}P < 0.01$. Source data are provided as a Source Data file.

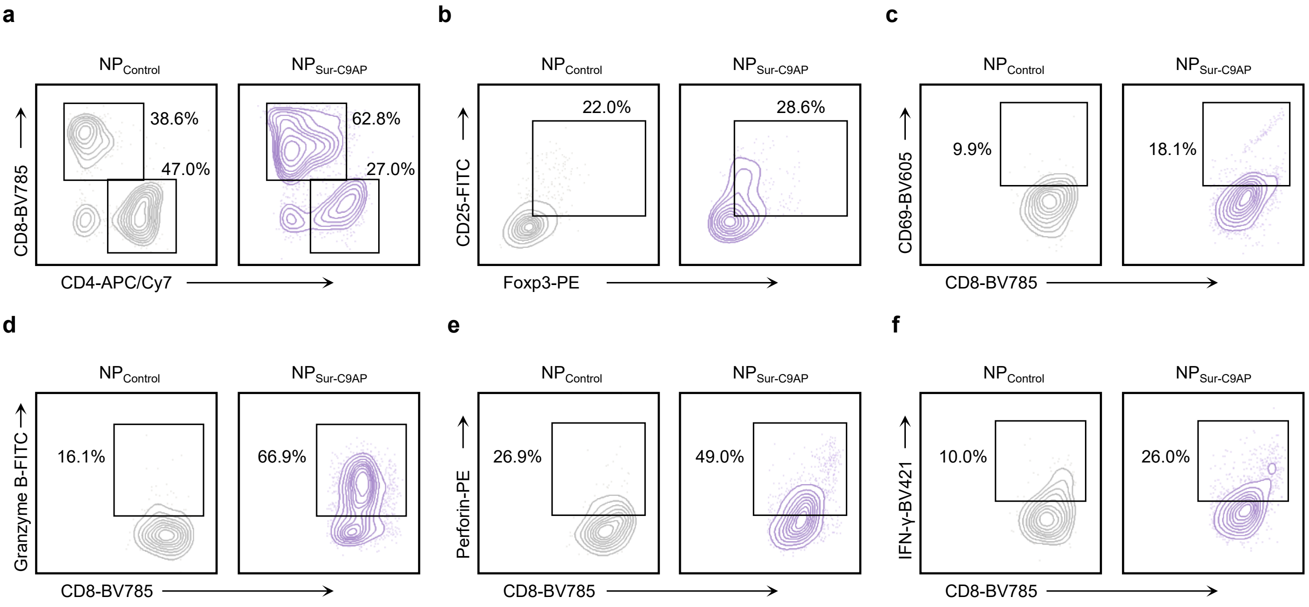

Supplementary Figure 31 Representative flow cytometry plots of CD8$^+$ T cells, CD69-positive CD8$^+$ T cells, Tregs and granzyme B-, perforin-, IFN-γ-positive CD8$^+$ T cells in B16-F10 melanoma after NP$_{Sur\text{-}C9AP}$ treatments. a Representative flow cytometry plots of CD8$^+$ T cell percentages in CD3$^+$ T cells of B16-F10 melanoma. **b** Representative flow cytometry plots of Treg percentages in CD4$^+$ T cells of B16-F10 melanoma. **c** to **f** CD69- (**c**), granzyme B- (**d**), perforin- (**e**) and IFN-γ- (**f**) positive cell percentages in CD8$^+$ T cells of B16-F10 melanoma. The statistical data were correspondingly shown in Fig. 8h to l.

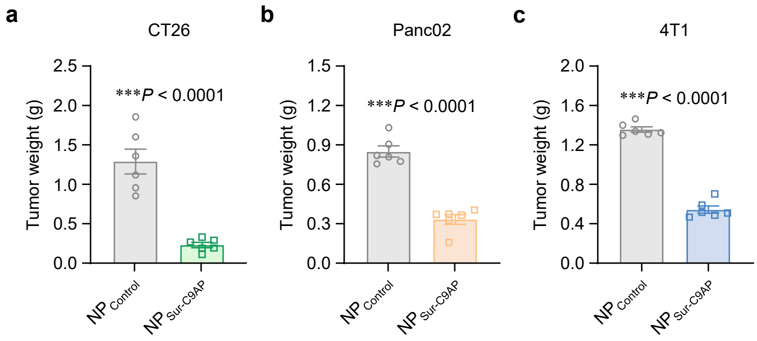

Supplementary Figure 32 Final tumor weights of CT26 (a), Panc02 (b) and 4T1 (c) tumor-bearing mice after $NP_{Sur\text{-}C9AP}$ treatments. Mice bearing CT26, Panc02 or 4T1 tumors were intravenously injected with $NP_{Control}$ or $NP_{Sur\text{-}C9AP}$ every other day for five injections (n = 6 mice per group). The injection doses were 1 mg pUC57 or pSur-C9AP per kg body weight. The final tumor weights of the tumor-bearing mice were measured at the end of treatments. The data are shown as the means ± SEM of n = 6 biologically independent mice. Statistical data were analyzed by two-sided student's t-test. ***$P < 0.001$. Source data are provided as a Source Data file.

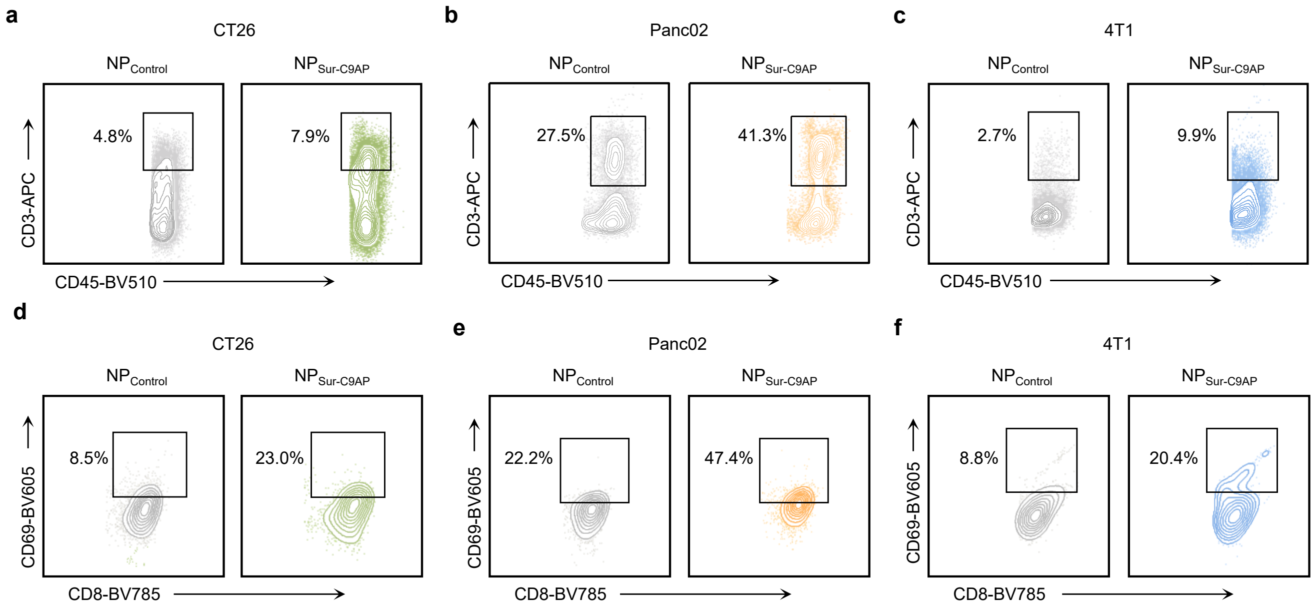

Supplementary Figure 33 Representative flow cytometry plots of CD3$^+$ T cells and CD69-positive CD8$^+$ T cells in different tumors after NP$_{\text{Sur-C9AP}}$ treatments. a to **c** Representative flow cytometry plots of CD3$^+$ T cell percentages in CD45$^+$ cells of CT26 (**a**), Panc02 (**b**) or 4T1 (**c**) tumors. **d** to **f** Representative flow cytometry plots of CD69-positive cell percentages in CD8$^+$ T cells of CT26 (**d**), Panc02 (**e**) or 4T1 (**f**) tumors. The statistical data were correspondingly shown in Fig. 8p to u.

Supplementary Table 1 DNA sequences of Tyr promoter, Sur promoter, CXCL9 and αPD-L1.

Tyr promoter (546 bps)	tctgcaggtcatagttcctgccagctgactttgtcaagacagtgatgtctgtgttccagcagttgttctgagt<br>atccttttcattatccactgtcctttcttcttaaattccacccccaacattgtaaatagcttctttcttaaactctgt<br>tcaaagaaccagcttgagtgtgtcagctgcttcctgctggggtcctggcaaaccactagtgacctttattc<br>ataagagatgatgtattcttgatactacttctcatttgcaaattccaattattattaatttcatatcaattagaata<br>atatatcttccttcaatttagttacctcactatgggctatgtacaaactccaagaaaaagttagtcatgtgcttt<br>gcagaagataaaagcttagtgtaaaacaggctgagagtatttgatgtaagaaggggagtggttatatag<br>gtcttagccaaaacatgtgatagtcactccaggggttgctggaaaagaagtctgtgacactcattaaccta<br>ttggtgcagattttgtatgatctaaaggagac
Sur promoter (260 bps)	ggcagggacgagctggcgcggcgtcgctgggtgcaccgcgaccacgggcagagccacgcggcgg<br>gaggactacaactcccggcacaccccgcgccgccccgcctctactcccagaaggccgcggggggtg<br>gaccgcctaagagggcgtgcgctcccgacatgccccgcggcgcgccattaaccgccagatttgaatc<br>gcgggacccgttggcagaggtggcggcggcggcatgggtgccccgacgttgccccctgcctgg
CXCL9 (378 bps)	atgaagtccgctgttcttttcctcttgggcatcatcttcctggagcagtgtggagttcgaggaaccctagtg<br>ataaggaatgcacgatgctcctgcatcagcaccagccgaggcacgatccactacaaatccctcaaaga<br>cctcaaacagtttgccccaagccccaattgcaacaaaactgaaatcattgctacactgaagaacggaga<br>tcaaacctgcctagatccggactcggcaaatgtgaagaagctgatgaaagaatgggaaaagaagatca<br>gccaaaagaaaaagcaaaagagggggaaaaaacatcaaaagaacatgaaaaacagaaaacccaaa<br>acaccccaaagtcgtcgtcgttcaaggaagactaca
αPD-L1 (795 bps)	atggaaaccgataccctgctgctgtgggtgctgctgctgtgggtgccgggcagcaccggcgatgatatt<br>cagatgacccagagcccgagcagcctgagcgcgagcgtgggcgatcgcgtgaccattacctgccgc<br>gcgagccaggatgtgagcaccgcggtggcgtggtatcagcagaaaccgggcaaagcgccgaaact<br>gctgatttatagcgcgagctttctgtatagcggcgtgccgagccgctttagcggcagcggcagcggcac<br>cgattttaccctgaccattagcagcctgcagccggaagattttgcgacctattattgccagcagtatctgta<br>tcatccggcgacctttggccagggcaccaaagtggaaattaaacgcggaggcggtggttccggcgga<br>ggaggcagcggtggcggaggttccgaagtgcagctggtggaaagcggcggcggcctggtgcagcc<br>gggcggcagcctgcgcctgagctgcgcggcgagcggctttacctttagcgatagctggattcattgggt<br>gcgccaggcgccgggcaaaggcctggaatgggtggcgtggattagcccgtatggcggcagcaccta<br>ttatgcggatagcgtgaaaggccgctttaccattagcgcggataccagcaaaaacaccgcgtatctgca<br>gatgaacagcctgcgcgcggaagataccgcggtgtattattgcgcgcgccgccattggccgggcggc<br>tttgattattggggccagggcaccctggtgaccgtgagcgcgctgagcagc

Supplementary Table 2 qRT-PCR primer sequences for analyzing the expression of CXCL9 and αPD-L1.

Primer ID	Sequence
CXCL9 forward primer	5'-TCCTCTTGGGCATCATCTTCC-3'
CXCL9 reverse primer	5'-TTTGTAGTGGATCGTGCCTCG-3'
αPD-L1 forward primer	5'-GAATGGGTGGCGTGGATTAG-3'
αPD-L1 reverse primer	5'-GGCTGTTCATCTGCAGATACG-3'
GAPDH forward primer	5'-AGGTCGGTGTGAACGGATTTG-3'
GAPDH reverse primer	5'-TGTAGACCATGTAGTTGAGGTCA-3'

Supplementary Table 3 qRT-PCR primer sequences for analyzing the expression of 5 key cytokines.

Primer ID	Sequence
IFN-γ forward primer	5'-ATGAACGCTACACACTGCATC-3'
IFN-γ reverse primer	5'-CCATCCTTTTGCCAGTTCCTC-3'
IL-6 forward primer	5'-TAGTCCTTCCTACCCCAATTTCC-3'
IL-6 reverse primer	5'-TTGGTCCTTAGCCACTCCTTC-3'
GM-CSF forward primer	5'-GGCCTTGGAAGCATGTAGAGG-3'
GM-CSF reverse primer	5'-GGAGAACTCGTTAGAGACGACTT-3'
IL-4 forward primer	5'-GGTCTCAACCCCCAGCTAGT-3'
IL-4 reverse primer	5'-GCCGATGATCTCTCTCAAGTGAT-3'
IL-10 forward primer	5'-GCTCTTACTGACTGGCATGAG-3'
IL-10 reverse primer	5'-CGCAGCTCTAGGAGCATGTG-3'
GAPDH forward primer	5'-AGGTCGGTGTGAACGGATTTG-3'
GAPDH reverse primer	5'-TGTAGACCATGTAGTTGAGGTCA-3'

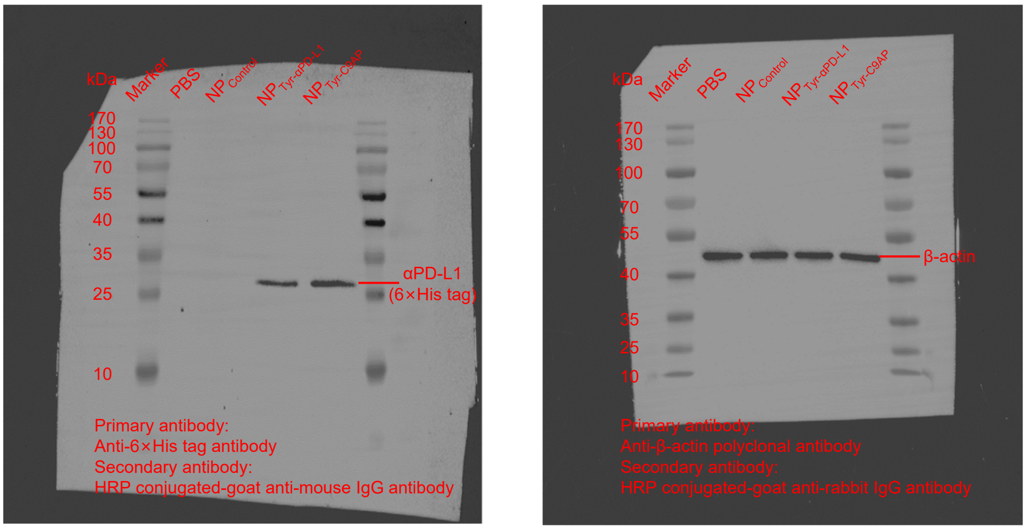

Supplementary Figure 7

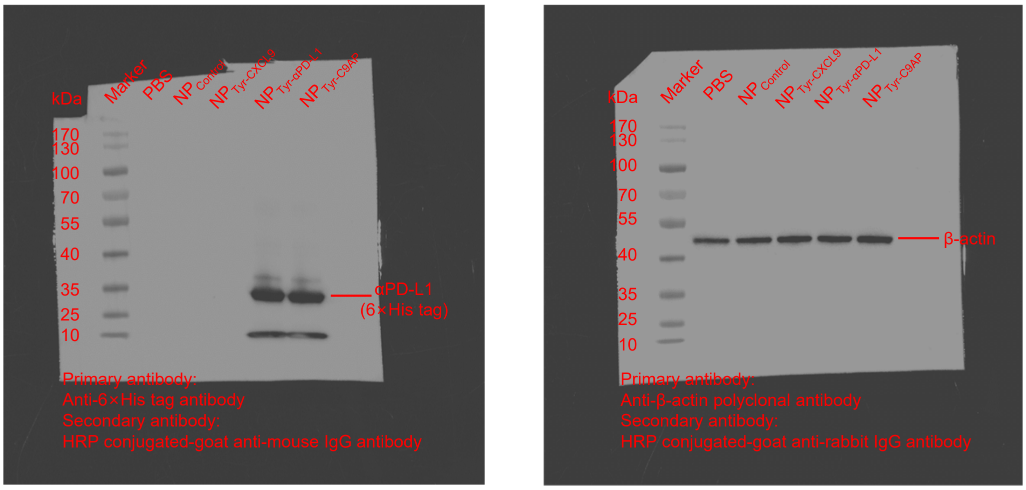

Supplementary Figure 12

Supplementary Figure 34 Scans of the films used to generate western blot data for Supplementary Figure 7 and Supplementary Figure 12.
