# Supplementary material for: Engineering tumor-specific gene nanomedicine to recruit and activate T cells for enhanced immunotherapy
Source: Nat Commun. 2023 Apr 8;14:1993. doi: 10.1038/s41467-023-37656-w (PMC10082825; doi:10.1038/s41467-023-37656-w)
Supplement: Supplementary file 3 — Reporting Summary [file 41467_2023_37656_MOESM3_ESM.pdf]

nature portfolio

Corresponding author(s): Jun Wang and Cong-Fei Xu

Last updated by author(s): Mar 12, 2023

Reporting Summary

Nature Portfolio wishes to improve the reproducibility of the work that we publish. This form provides structure for consistency and transparency in reporting. For further information on Nature Portfolio policies, see our Editorial Policies and the Editorial Policy Checklist.

Statistics

For all statistical analyses, confirm that the following items are present in the figure legend, table legend, main text, or Methods section.

n/a	Confirmed	
☐	☒	The exact sample size (*n*) for each experimental group/condition, given as a discrete number and unit of measurement
☐	☒	A statement on whether measurements were taken from distinct samples or whether the same sample was measured repeatedly
☐	☒	The statistical test(s) used AND whether they are one- or two-sided<br>*Only common tests should be described solely by name; describe more complex techniques in the Methods section.*
☒	☐	A description of all covariates tested
☐	☒	A description of any assumptions or corrections, such as tests of normality and adjustment for multiple comparisons
☐	☒	A full description of the statistical parameters including central tendency (e.g. means) or other basic estimates (e.g. regression coefficient) AND variation (e.g. standard deviation) or associated estimates of uncertainty (e.g. confidence intervals)
☐	☒	For null hypothesis testing, the test statistic (e.g. *F*, *t*, *r*) with confidence intervals, effect sizes, degrees of freedom and *P* value noted<br>*Give P values as exact values whenever suitable.*
☒	☐	For Bayesian analysis, information on the choice of priors and Markov chain Monte Carlo settings
☒	☐	For hierarchical and complex designs, identification of the appropriate level for tests and full reporting of outcomes
☒	☐	Estimates of effect sizes (e.g. Cohen's *d*, Pearson's *r*), indicating how they were calculated

Our web collection on statistics for biologists contains articles on many of the points above.

Software and code

Policy information about availability of computer code

Data collection	BD FACS Diva software v8.0.1.1 (flow cytometry)<br>ZEISS ZEN2 (black edition) software (confocal)<br>Living image software v4.4 (in vivo imaging)<br>NIS-Elements Viewer software v5.21 (fluorescence imaging)<br>Digital Pathology Scanner software (histopathology)
Data analysis	FlowJo software v10.0.7 (flow cytometry)<br>ZEISS ZEN2 (blue edition) software (confocal)<br>Graphpad Prism v8.0 (statistical analysis)

For manuscripts utilizing custom algorithms or software that are central to the research but not yet described in published literature, software must be made available to editors and reviewers. We strongly encourage code deposition in a community repository (e.g. GitHub). See the Nature Portfolio guidelines for submitting code & software for further information.

Data

Policy information about availability of data

All manuscripts must include a data availability statement. This statement should provide the following information, where applicable:

- Accession codes, unique identifiers, or web links for publicly available datasets
- A description of any restrictions on data availability
- For clinical datasets or third party data, please ensure that the statement adheres to our policy

The sequence of CXCL9 cDNA was obtained from the CCDS database with ID number CCDS39152.1 (https://www.ncbi.nlm.nih.gov/projects/CCDS/CcdsBrowse.cgi). The data that support this study are available within the article andArticle, its Supplementary Information or Source data files or available from the authors upon request. Source data are provided with this paper.

Human research participants

Policy information about studies involving human research participants and Sex and Gender in Research.

Reporting on sex and gender	N/A
Population characteristics	N/A
Recruitment	N/A
Ethics oversight	N/A

Note that full information on the approval of the study protocol must also be provided in the manuscript.

Field-specific reporting

Please select the one below that is the best fit for your research. If you are not sure, read the appropriate sections before making your selection.

☒ Life sciences ☐ Behavioural & social sciences ☐ Ecological, evolutionary & environmental sciences

For a reference copy of the document with all sections, see nature.com/documents/nr-reporting-summary-flat.pdf

Life sciences study design

All studies must disclose on these points even when the disclosure is negative.

Sample size	All in vitro data were done at least in triplicate and SEM were reported as stated in the figure and figure legends. For in vivo experiments, 3 to 10 mice were sufficient to identify differences between groups with at least 80% power and a 5% significance level. Sample size was estimated on the basis of similar research reported in the literature (references below). Details regarding sample size of all experiments are provided in the Methods section and figure legends.<br>1. Y. Zhai, et al. T lymphocyte membrane-decorated epigenetic nanoinducer of interferons for cancer immunotherapy. Nat Nanotechnol. 2021 Nov;16(11):1271-1280.<br>2. C. Liu, et al. A nanovaccine for antigen self-presentation and immunosuppression reversal as a personalized cancer immunotherapy strategy. Nat Nanotechnol. 2022 May;17(5):531-540.
Data exclusions	No data were excluded from the analysis.
Replication	All in vitro cell assays included 3-4 biologically independent samples or independent experiments. All reported attempts at replication were successful. For in vivo studies, each treatment group consisted of 4-10 mice. In vivo studies consisted of at least a preliminary experiment and a formal treatment experiment.
Randomization	All samples, cells and mice were allocated randomly into experimental groups.
Blinding	No formal blinding was used. In vitro treatments were not blinded since each experiment was typically conducted/analyzed by a single investigator. The investigator organizing the experimental groups and involved in sample collection was not blinded; however, colleagues aiding in data collection were blinded. The investigators were blinded to group allocation during downstream assays.

Reporting for specific materials, systems and methods

We require information from authors about some types of materials, experimental systems and methods used in many studies. Here, indicate whether each material, system or method listed is relevant to your study. If you are not sure if a list item applies to your research, read the appropriate section before selecting a response.

Materials & experimental systems

n/a	Involved in the study
☐	☒ Antibodies
☐	☒ Eukaryotic cell lines
☒	☐ Palaeontology and archaeology
☐	☒ Animals and other organisms
☒	☐ Clinical data
☒	☐ Dual use research of concern

Methods

n/a	Involved in the study
☒	☐ ChIP-seq
☐	☒ Flow cytometry
☒	☐ MRI-based neuroimaging

Antibodies

Antibodies used

The following primary antibodies were used for flow cytometry. They are listed as antigen first, following by supplier, catalog number and clone/lot number as applicable. All surface antibodies were used at 1:100 dilution; all intracellular antibodies were used at 1:50 dilution.
1. Purified anti-mouse CD16/32 Antibody, Biolegend, cat. no. 101302, Clone: 93;
2. Brilliant Violet 510™ anti-mouse CD45 Antibody, Biolegend, cat. no. 103137, Clone: 30-F11;
3. APC anti-mouse CD3 Antibody, Biolegend, cat. no. 100311, Clone: 145-2C11;
4. APC/Cyanine7 anti-mouse CD4 Antibody, Biolegend, cat. no. 100413, Clone: GK1.5;
5. Brilliant Violet 785 anti-mouse CD8a, Biolegend, cat. no. 100749, Clone: 53-6.7;
6. FITC anti-mouse CD8a Antibody, Biolegend, cat. no. 100705, Clone: 53-6.7;
7. Brilliant Violet 605 anti-mouse CD69 Antibody, Biolegend, cat. no. 104529, Clone:H1.2F3;
8. Alexa Fluor 488 anti-mouse NK1.1 Antibody, Biolegend, cat. no.108717, Clone: PK136;
9. FITC anti-mouse NKp46 Antibody, Biolegend, cat. no. 137605, Clone: 29A1.4;
10. FITC anti-mouse CD11b Antibody, Biolegend, cat. no. 101205, Clone: M1/70;
11. APC/Cyanine7 anti-mouse Ly-6G/Ly-6C (Gr-1) Antibody, Biolegend, cat. no. 108423, Clone: RB6-8C5;
12. Brilliant Violet 650 anti-mouse F4/80 Antibody, Biolegend, cat. no.123149, Clone: BM8;
13. PE/Dazzle594 anti-mouse CD80 Antibody, Biolegend, cat. no. 104737, Clone: 16-10A1;
14. PE anti-mouse CD206 Antibody, Biolegend, cat. no.141705, Clone: C068C2;
15. FITC anti-mouse CD25 Antibody, Biolegend, cat. no. 101907, Clone: 3C7;
16. PE anti-mouse Foxp3 Antibody, Biolegend, cat. no.126403, Clone: MF-14;
17. Brilliant Violet 421 anti-mouse IFN-γ Antibody, Biolegend, cat. no. 505829, Clone: XMG1.2;
18. FITC anti-human/mouse Granzyme B Recombinant Antibody, Biolegend, cat. no. 396404, Clone: QA16A02;
19. PE anti-mouse Perforin Antibody, Biolegend, cat. no.154406, Clone: S16009B;
20. Alexa Fluor 647 anti-6×His Tag Antibody, Biolegend, cat.no.652513, Clone: J099B12.

The following primary antibodies were used for Western Blot, Immunohistochemical staining. They are listed as antigen first, following by supplier, catalog number and clone/lot number as applicable.
1. Anti-6×His tag Antibody, Abcam, cat. no. ab18184, Clone: HIS.H8, dilution 1:10000;
2. Anti-β-actin Polyclonal Antibody, Biosharp, cat. no. BL005B, dilution 1:4000;
3. HRP conjugated-goat anti-mouse IgG Antibody, Biosharp, cat. no. BL001A, dilution 1:5000;
4. HRP conjugated-goat anti-rabbit IgG Antibody, Biosharp, cat. no. BL003A, dilution 1:5000;
5. Anti-CD3ε Rabbit mAb, Cell Signaling Technology, cat.no.99940T, Clone: D4V8L, dilution 1:200;
6. Anti-CD8α Rabbit mAb, Cell Signaling Technology, cat.no.98941T, Clone: D4W2Z, dilution 1:200;

The following primary antibodies were used for in vivo therapy and ELISA.
1. InVivoMAb anti-mouse PD-L1 (B7-H1), BioxCell, cat.no.BE0101, Clone:10F.9G2, working concentration 1 mg ml-1;
2. HRP conjugated-anti-His tag antibody, SinoBiological, cat.no. 105327-MM02T-H, working concentration 1 μg ml-1.

Validation

All antibodies were verified by the supplier and each lot has been quality tested. All validation statements can be found on the respective antibody website:
1. Purified anti-mouse CD16/32 Antibody: https://www.biolegend.com/en-us/search-results/purified-anti-mouse-cd16-32-antibody-190;
2. Brilliant Violet 510™ anti-mouse CD45 Antibody: https://www.biolegend.com/en-us/products/brilliant-violet-510-anti-mousecd45-antibody-7995;
3. APC anti-mouse CD3 Antibody:
https://www.biolegend.com/en-us/products/apc-anti-mouse-cd3epsilon-antibody-21;
4. APC/Cyanine7 anti-mouse CD4 Antibody:
https://www.biolegend.com/en-us/products/apc-cyanine7-anti-mouse-cd4-antibody-1964;
5. Brilliant Violet 785 anti-mouse CD8a Antibody:

https://www.biolegend.com/en-us/products/brilliant-violet-785-anti-mouse-cd8a-antibody-7957;
6. FITC anti-mouse CD8a Antibody:
https://www.biolegend.com/en-us/products/fitc-anti-mouse-cd8a-antibody-153;
7. Brilliant Violet 605 anti-mouse CD69 Antibody:
https://www.biolegend.com/en-us/products/brilliant-violet-605-anti-mouse-cd69-antibody-7864;
8. Alexa Fluor 488 anti-mouse NK1.1 Antibody:
https://www.biolegend.com/en-us/products/alexa-fluor-488-anti-mouse-nk-1-1-antibody-3143;
9. FITC anti-mouse NKp46 Antibody:
https://www.biolegend.com/en-us/products/fitc-anti-mouse-cd335-nkp46-antibody-6618;
10. FITC anti-mouse CD11b Antobody:
https://www.biolegend.com/en-us/products/fitc-anti-mouse-human-cd11b-antibody-347;
11. APC/Cyanine7 anti-mouse Ly-6G/Ly-6C (Gr-1) Antibody:
https://www.biolegend.com/en-us/products/apc-cyanine7-anti-mouse-ly-6gly-6c-gr-1-antibody-3935;
12. Brilliant Violet 650 anti-mouse F4/80 Antibody:
https://www.biolegend.com/en-us/products/brilliant-violet-650-anti-mouse-f4-80-antibody-10630;
13. PE/Dazzle594 anti-mouse CD80 Antibody:
https://www.biolegend.com/en-us/products/pe-dazzle-594-anti-mouse-cd80-antibody-10221;
14. PE anti-mouse CD206 Antibody:
https://www.biolegend.com/en-us/products/pe-anti-mouse-cd206-mmr-antibody-7424;
15. FITC anti-mouse CD25 Antibody:
https://www.biolegend.com/en-us/products/fitc-anti-mouse-cd25-antibody-4511;
16. PE anti-mouse Foxp3 Antibody:
https://www.biolegend.com/en-us/products/pe-anti-mouse-foxp3-antibody-4660;
17. Brilliant Violet 421 anti-mouse IFN-γ Antibody:
https://www.biolegend.com/en-us/products/brilliant-violet-421-anti-mouse-ifn-gamma-antibody-7154;
18. FITC anti-human/mouse Granzyme B Recombinant Antibody:
https://www.biolegend.com/en-us/products/fitc-anti-human-mouse-granzyme-b-recombinant-antibody-17399;
19. PE anti-mouse Perforin Antibody:
https://www.biolegend.com/en-us/products/pe-anti-mouse-perforin-antibody-15255;
20. Alexa Fluor 647 anti-6×His Tag Antibody:
https://www.biolegend.com/en-us/products/alexa-fluor-647-anti-his-tag-antibody-14991?GroupID=BLG13798
21. Anti-6×His tag Antibody:
https://www.abcam.cn/6x-his-tag-antibody-hish8-ab18184.html;
22. Anti-β-actin Polyclonal Antibody:
http://biosharp.cn/index/product/details/language/cn/product_id/92.html;
23. HRP conjugated-goat anti-mouse IgG Antibody:
http://biosharp.cn/index/product/details/language/cn/product_id/78.html;
24. HRP conjugated-goat anti-rabbit IgG Antibody:
http://biosharp.cn/index/product/details/language/cn/product_id/90.html;
25. Anti-CD3ε Rabbit mAb:
https://www.cellsignal.com/products/primary-antibodies/cd3e-d4v8l-rabbit-mab/99940;
26. Anti-CD8α Rabbit mAb:
https://www.cellsignal.com/products/primary-antibodies/cd8a-d4w2z-xp-rabbit-mab-mouse-specific/98941;
27. InVivoMAb anti-mouse PD-L1 (B7-H1):
https://bioxcell.com/invivomab-anti-mouse-pd-l1-b7-h1-be0101;
28. HRP conjugated-anti-His tag antibody:
https://cn.sinobiological.com/antibodies/his-tag-105327-mm02t-h.

Eukaryotic cell lines

Policy information about cell lines and Sex and Gender in Research

Cell line source(s)	The mouse cell lines, including B16-F10, CT26, Panc02, 4T1, C2C12, NIH/3T3, DC2.4, RAW264.7, Clone M-3 (Cloudman S91) and YUMM1.7 cells were obtained from the American Type Culture Collection (ATCC). The B16-F10-OVA-EGFP cell line was constructed by infecting B16-F10 cells with a lentivirus encoding OVA and EGFP. The human cell line 293F was obtained from the National Collection of Authenticated Cell Cultures.
Authentication	Authentication was performed by ATCC for B16-F10, CT26, Panc02, 4T1, C2C12, NIH/3T3, DC2.4, RAW264.7, Clone M-3 (Cloudman S91), YUMM1.7 cell lines and by National Collection of Authenticated Cell Cultures for 293F cell line (Method: STR profiling).
Mycoplasma contamination	All cell lines were tested for mycoplasma contamination. No mycoplasma contamination was found.
Commonly misidentified lines (See ICLAC register)	No commonly misidentified cell lines were used in the study.

Animals and other research organisms

Policy information about studies involving animals; ARRIVE guidelines recommended for reporting animal research, and Sex and Gender in Research

Laboratory animals	Female C57BL/6 (6-10 weeks) and BALB/c (6-10 weeks) mice were purchased from Silaike Jingda Laboratory Animal Co., Ltd. (Hunan, China). OT-1 transgenic mice (C57BL/6-Tg (TcraTcrb)1100Mjb/J) (6-10 weeks) were gifted from Prof. Tian-Meng Sun of Jilin University. All mice were maintained at the specific pathogen-free (SPF) facility of the South China University of Technology (SCUT) SCUT and received care in compliance with the Guide for the Care and Use of Laboratory Animals. All mice were housed in temperatures 20-25°C, humidity 30-70% and a 12 h light/12 h dark cycle.
Wild animals	The study did not involve wild animals.
Reporting on sex	This finding applies to both sexes.
Field-collected samples	The study did not involve samples collected from the field.
Ethics oversight	All the animal experiments were approved by the Animal Care and Use Committee at South China University of Technology (SCUT), and every effort was made to minimize suffering from experiments (official approval number: 2019012).

Note that full information on the approval of the study protocol must also be provided in the manuscript.

Flow Cytometry

Plots

Confirm that:

- [x] The axis labels state the marker and fluorochrome used (e.g. CD4-FITC).
- [x] The axis scales are clearly visible. Include numbers along axes only for bottom left plot of group (a 'group' is an analysis of identical markers).
- [x] All plots are contour plots with outliers or pseudocolor plots.
- [x] A numerical value for number of cells or percentage (with statistics) is provided.

Methodology

Sample preparation	At the end of tumor treatment, the mice were sacrificed, and the tumor tissues or major organs (heart, liver, spleen, lung, kidney) were collected. The tumor tissues were dissociated into single cells by enzymatic digestion in RPMI 1640 medium containing type-IV collagenase (1 mg/mL), deoxyribonuclease I (100 µg/mL)and hyaluronidase (100 µg/mL) at 37°C for 25 min. Digested cells were passed through a 40-µm nylon mesh and collected by centrifugation at 1,500 revolutions per minute for 10 min, followed by Red Blood Cell (RBC) lysis. 100 µL of cell suspension (20 million cells/mL) was used for flow cytometry detection.
Instrument	FACSCelesta flow cytometer
Software	Data collection: BD FACS Diva software v8.0.1.1<br>Data anlysis: FlowJo software v10.0.7
Cell population abundance	No sorting was performed.
Gating strategy	The detailed gating strategy could be found in the Supplementary Figure 17.a Gating strategies of CD3+, CD4+, CD8+, CD69-positive CD8+ T cells and natural killer (NK) cells. b Gating strategies of myeloid-derived suppressor cells (MDSCs), M1-like and M2-like macrophages (MΦ). c Gating strategies of granzyme B-, perforin- and IFN-γ-positive CD8+ T cells. d Gating strategies of regulatory T cells (Tregs).

- [x] Tick this box to confirm that a figure exemplifying the gating strategy is provided in the Supplementary Information.
